# First Characterization of *Ostreopsis* cf. *ovata* (Dinophyceae) and Detection of Ovatoxins during a Multispecific and Toxic *Ostreopsis* Bloom on French Atlantic Coast

**DOI:** 10.3390/md20070461

**Published:** 2022-07-18

**Authors:** Nicolas Chomérat, Elvire Antajan, Isabelle Auby, Gwenael Bilien, Liliane Carpentier, Marie-Noëlle de Casamajor, Florian Ganthy, Fabienne Hervé, Magali Labadie, Claire Méteigner, Camille Paradis, Myriam Perrière-Rumèbe, Florence Sanchez, Véronique Séchet, Zouher Amzil

**Affiliations:** 1 Ifremer, LITTORAL, 29900 Concarneau, France; gwenael.bilien@ifremer.fr; 2 Ifremer, LITTORAL, 33120 Arcachon, France; elvire.antajan@ifremer.fr (E.A.); isabelle.auby@ifremer.fr (I.A.); florian.ganthy@ifremer.fr (F.G.); claire.meteigner@ifremer.fr (C.M.); myriam.rumebe@ifremer.fr (M.P.-R.); 3 Ifremer, PHYTOX, 44000 Nantes, France; liliane.carpentier@ifremer.fr (L.C.); fabienne.herve@ifremer.fr (F.H.); veronique.sechet@ifremer.fr (V.S.); 4 Ifremer, LITTORAL, 64600 Anglet, France; marie.noelle.de.casamajor@ifremer.fr (M.-N.d.C.); florence.sanchez@ifremer.fr (F.S.); 5 Centre Anti-Poison et Toxicovigilance, CHU de Bordeaux, 33076 Bordeaux, France; magali.labadie@chu-bordeaux.fr (M.L.); camille.paradis@chu-bordeaux.fr (C.P.)

**Keywords:** *Ostreopsis* cf. *ovata*, ovatoxins, intoxication, microscopy, phylogeny, LC–MS/MS, human respiratory symptoms

## Abstract

Blooms of the benthic toxic dinoflagellate genus *Ostreopsis* have been recorded more frequently during the last two decades, particularly in warm temperate areas such as the Mediterranean Sea. The proliferation of *Ostreopsis* species may cause deleterious effects on ecosystems and can impact human health through skin contact or aerosol inhalation. In the eastern Atlantic Ocean, the toxic *O.* cf. *ovata* has not yet been reported to the north of Portugal, and the only species present further north was *O.* cf. *siamensis*, for which the toxic risk is considered low. During summer blooms of unidentified *Ostreopsis* species on the French Basque coast (Atlantic) in 2020 and 2021, people suffered from irritations and respiratory disorders, and the number of analyzed cases reached 674 in 2021. In order to investigate the causes, sampling was carried out during summer 2021 to (i) taxonomically identify *Ostreopsis* species present using a molecular approach, (ii) isolate strains from the bloom and culture them, and (iii) characterize the presence of known toxins which may be involved. For the first time, this study reports the presence of both *O.* cf. *siamensis* and *O.* cf. *ovata*, for which the French Basque coast is a new upper distribution limit. Furthermore, the presence of ovatoxins a, b, c, and d in the environmental sample and in a cultivated strain in culture confirmed the toxic nature of the bloom and allowed identifying *O.* cf. *ovata* as the producer. The present data identify a new health risk in the area and highlight the extended distribution of some harmful dinoflagellates, presumably in relation to climate change.

## 1. Introduction

Primarily considered as a tropical dinoflagellate genus described from the Gulf of Thailand (Siam) [1], *Ostreopsis* has been more frequently reported in warm temperate areas, probably as a consequence of climate change in the past decades [2]. In the Mediterranean Sea, *Ostreopsis* has been mentioned since the 1970s, but it has become increasingly frequent with massive blooms, causing detrimental effects on marine benthic communities [3,4] and on human health through skin contact [5,6], toxic aerosols [7,8], and contaminated seafood [9,10].

To date, 11 species have been described and are taxonomically valid [11], but only two species include a morpho-molecular characterization [12,13]. While genus identification is relatively easy through light microscopy because of its typical teardrop shape, most of the species share rather similar morphological characters including thecal plate organization, and they can hardly be distinguished by light microscopy, especially when they co-occur [14,15,16]. Furthermore, a high degree of morphological variability exists within a given species, which makes the use of genetic methods essential for reliable identification at species level [17]. Phylogenetic studies inferred from ribosomal genes (domains of the large subunit, LSU) and intergenic transcribed spacers (ITS regions) revealed the existence of at least 13 major clades that could potentially correspond to different species [18,19,20]; however, to date, some of these genotypes have not yet been taxonomically identified owing to cryptic morphologies or a lack of morphological information [21,22]. Recent studies successfully clarified the taxonomic identity of some of these ribotypes [16,19,20,23], but such approaches are absolutely needed for the remaining genotypes to clarify the complex taxonomy of the genus.

The toxins produced by *Ostreopsis* species are analogues of palytoxin (PLTX), a highly potent marine toxin [24], first isolated from the zoanthid *Palythoa toxica* [25]. PLTX and its analogues may affect human health via ingestion of contaminated seafood, skin contact with seawater, and inhalation of marine aerosols [6,26,27,28,29]. Of the 11 taxonomically described species in the genus [11], the species *O. siamensis*, *O. mascarenensis*, *O.* cf. *ovata*, and *O. fattorussoi* produce some of the PLTX analogues (ovatoxins, ostreocins, and mascarenotoxins).

The ovatoxin group (OVTX) includes several analogues of PLTX, named after the organism *Ostreopsis* cf. *ovata* in which they have been primarily identified during toxic blooms in the Mediterranean Sea [30]. On the basis of profiles acquired by liquid chromatography coupled with high-resolution tandem mass spectrometry (LC–HRMS/MS), these compounds were identified as PLTX analogues, with ovatoxin a (OVTX-a), the first for which the structure was elucidated [30,31,32]. OVTX-a is structurally very similar to PLTX, but lacks two oxygen atoms. Since 2010, other analogues of the OVTX group have been characterized in Mediterranean strains of *O.* cf. *ovata*: OVTX-b, -c, -d, and -e [33], OVTX-f [34], OVTX-g [35], OVTX-h [36], and OVTX-l [37]. Except for OVTX-a, among these analogues, no complete chemical structure has been elucidated, and only fragmentations by HRMS allowed formulating structural hypotheses. Toxin profiles of Mediterranean strains of *O.* cf. *ovata* reported so far appear to be dominated by OVTX-a followed by OVTX-b, the isomers OVTX-d and -e, OVTX-c, and isobaric PLTX [37,38,39]. Other ovatoxins have been identified, e.g., OVTX-i, j1, j2, and k in *O. fattorussoi* isolated from Lebanese and Cypriot coasts [13,40]. Mediterranean OVTX isomers (OVTX-a, -b, -d, -e) have been identified in Japanese strains of *Ostreopsis* spp., named OVTX-a AC, -b AC, -d AC, and -e AC [41], as well as OVTX-a IK2, -d IK2, and -e IK2 [42].

In the tropical species *O. siamensis* and *O. mascarenensis*, ostreocins -A, -B, -D, and -E1 (OSTs) [43,44,45,46,47] and mascarenotoxins -a, -b, and -c (McTXs) [46,47,48] have been identified, respectively.

The majority of *Ostreopsis* poisonings are through inhalation, direct contact leading to skin irritation [6,48,49,50,51,52,53], or eye contact [52,54]. In temperate areas, toxic blooms accompanied by respiratory problems and skin irritation in humans exposed to marine aerosols containing algal toxins and/or cells debris and seawater [29] have been reported for several areas of the Mediterranean Sea in the two last decades [55]. Among the three species recorded so far in the Mediterranean Sea, *O.* cf. *ovata*, *O.* cf. *siamensis* now considered as clearly divergent from *O. siamensis* [18,19], and *O. fattorussoi*, the toxic effects have mainly been attributed to *O.* cf. *ovata*. Intoxication episodes associated with toxic blooms of *O.* cf. *ovata* occurred during the summer in the northwestern Mediterranean Sea, e.g., on the Italian coast of Genoa in 2005 and 2006 [56], Spanish coast in 2006 [57], and Algerian coast in 2009 [58]. In Genoa, more than 200 people near the beaches felt symptoms during the bloom of *O.* cf. *ovata* in 2005, among which 20 were hospitalized [56]. On the French Mediterranean coast, 47 intoxications were reported during bloom episodes between 2006 and 2009. The symptoms described correspond to skin irritations induced by direct contact with cells or mucus, along with irritations of the respiratory tract as well as nausea, fever, dizziness, coughing, and headaches induced by aerosols of *O.* cf. *ovata* [5].

In the western Atlantic Ocean, *Ostreopsis* outbreaks were also reported in western tropical parts of the Brazilian coasts [59,60,61], where both *O.* cf. *ovata* and *O.* cf. *siamensis* were present. In the eastern Atlantic, *Ostreopsis* spp. were detected on the Azores [62], the Canary Islands [59], Morocco [63], and southern Portugal, where *O.* cf. *siamensis* was first detected in 2008 and even bloomed in 2017 [64,65]. As reported in 2011, *O.* cf. *siamensis* was also present at several sites of the Cantabrian Sea (northern Spain) since 2007 [66]. While *O.* cf. *ovata* was later found in samples from Portugal [67,68,69], it has never been detected in northern places such as the Cantabrian and Basque coasts, where only *O.* cf. *siamensis* was shown to be present and considered to be the unique *Ostreopsis* species present in this area to date [70,71].

The present study reports atypical *Ostreopsis* bloom events at the end of summer 2020 and mid-summer 2021 on the French Basque coast, causing several health issues to surfers and swimmers, with about 700 people affected in 2021. This work aimed to identify the species present during the bloom using a genetic approach on isolated environmental cells and from cultivated strains, as well as characterize the toxin profile using LC–MS/MS.

## 2. Results

### 2.1. Respiratory Disorders and Cutaneous Irritations Observed during Summer Blooms on the French Basque Coast

**Figure 1:**
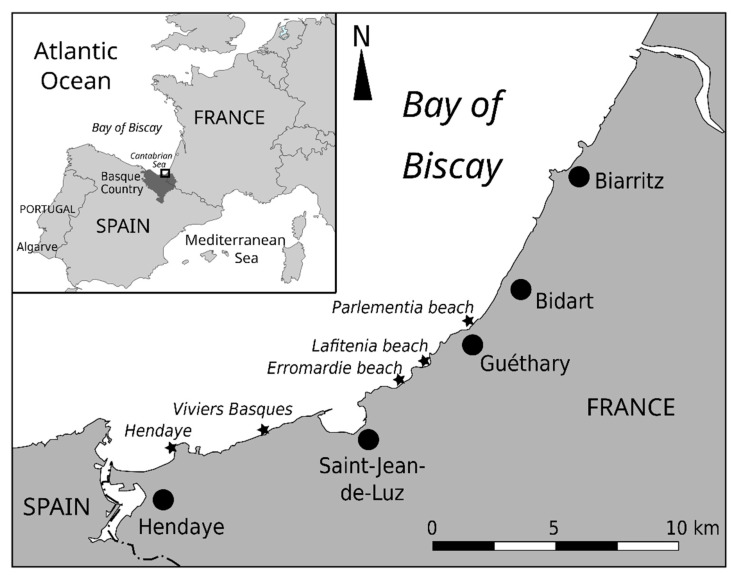
Map showing the location of Basque country and sites on the French Basque coast where proliferations of *Ostreopsis* spp. were observed in summers of 2020 and 2021 (stars).

In 2020, for the first time, a few reports of respiratory symptoms (about 10) were mentioned by swimmers and people frequenting beaches of Hendaye on the French Basque coast (between 4 September and 13 September), and numerous *Ostreopsis* cells were identified on a macroalgal sample collected at Hendaye (Figure 1). Nevertheless, because the number of reported cases remained low, and since it occurred at the end of summer, no further investigations of the origin of this toxicity were conducted. Since *O.* cf. *siamensis* was known to be recurrently present in the area, these effects were attributed to this species without any complementary analysis.

In 2021, an unusual outbreak of *Ostreopsis* starting in early August on some beaches of the Basque coast (Erromardie and Lafitenia beaches, near Saint-Jean-de-Luz, Figure 1) was associated with health disorders, and a great number of people reported symptoms. From a total of 830 reports of health disorders, after removal of duplicates, 674 cases were verified and confirmed by the Poison Control Center of the university hospital in Bordeaux. Beach users reported various respiratory and cutaneous symptoms (difficulty breathing, irritations, headaches, dry cough, nose irritation, eye irritation and/or dermatitis, and general malaise) after direct contact with water and/or after inhalation of marine aerosols. In addition, metallic taste has also been reported. Several health issues were also mentioned by people who visited beaches of other close localities such as Hendaye and Biarritz (Figure 1). Furthermore, closures of some beaches were decided by local authorities to prevent intoxications. The symptoms disappeared within 2 days without a need to medicate when people moved away from the area concerned by the *Ostreopsis* bloom.

Because of the importance of the phenomenon and number of affected people in summer 2021, a complete investigation of this toxic event was realized on the basis of regular sampling.

**Figure 2:**
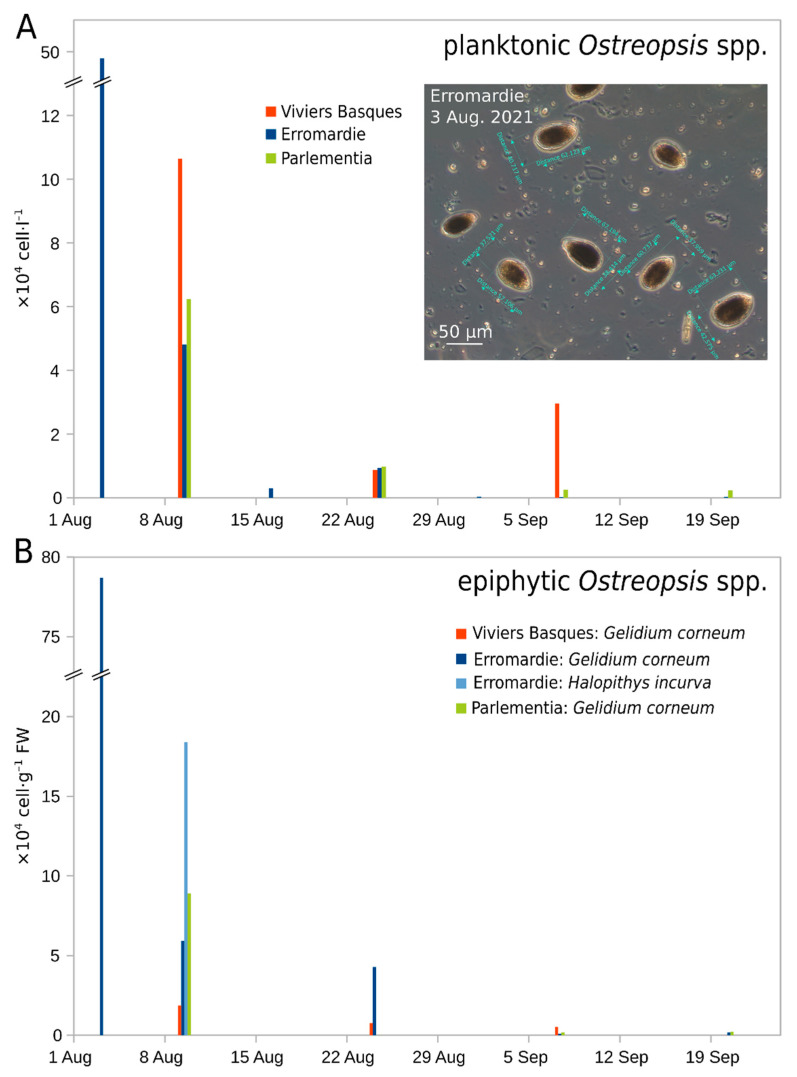
Abundances of *Ostreopsis* cells ((**A**), upper panel) and macroalgae ((**B**), lower panel) during summer 2021. The insert in (**A**) shows a micrograph of the Erromardie bloom water sample (3 August 2021) with many *Ostreopsis* cells fixed in Lugol’s solution.

**Figure 3:**
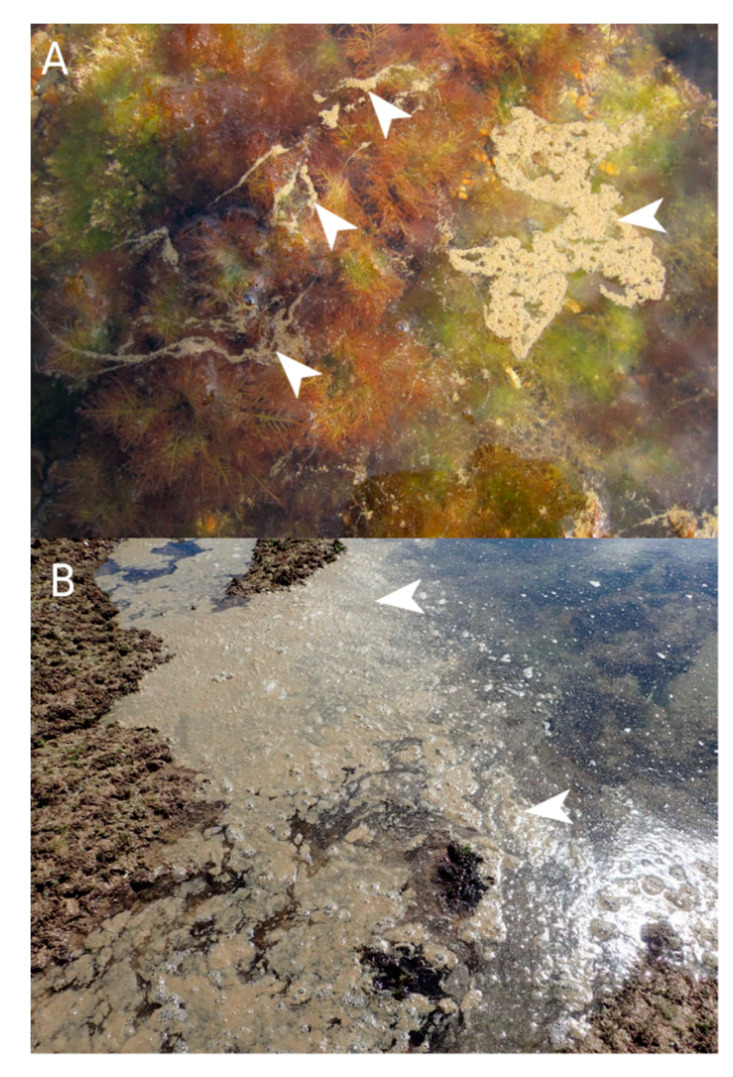
Scum-like aggregates (arrowheads) containing high concentration of *Ostreopsis* cells occasionally visible on the water surface in areas covered by macroalgae ((**A**), Alcyons beach, Guétary, 13 September 2021) or rocks ((**B**), Marbella beach, Biarritz, 10 September 2021).

Initial results of *Ostreopsis* cell counts from early August revealed abundances of about 50 × 10^4^ cells·L^−1^ in water samples and 80 × 10^4^ cells·g^−1^ FW of macroalgae (fresh weight *Gelidium corneum*) at Erromardie (Figure 2). In samples collected after 8 August and in September, cell concentrations decreased at the three investigated sites (Figure 2) indicating a decline in the bloom, and abundances were low at the end of September. During September, at some other localities (Guétary and Biarritz), visible aggregates containing high cell concentrations of cells were occasionally observed forming a scum-like accumulation on the surface of the water in areas containing macroalgae or rocks (Figure 3).

### 2.2. Environmental Conditions before and during the 2020 and 2021 Events

As the intoxication phenomenon of beach users occurred twice, in the summers 2020 and 2021, albeit to a lower extent in 2020, it seemed worthwhile presenting here the environmental conditions for both years. Results of the meteorological and oceanographic parameters are shown using a timescale corresponding to the date of occurrence of the health disorders, where the date “0” corresponds to the day on which the reports of sickness began (4 September 2020 and 3 August 2021). Hence, this representation makes it possible to compare the environmental conditions during the weeks preceding the onset of the intoxication episodes of both years.

**Figure 4:**
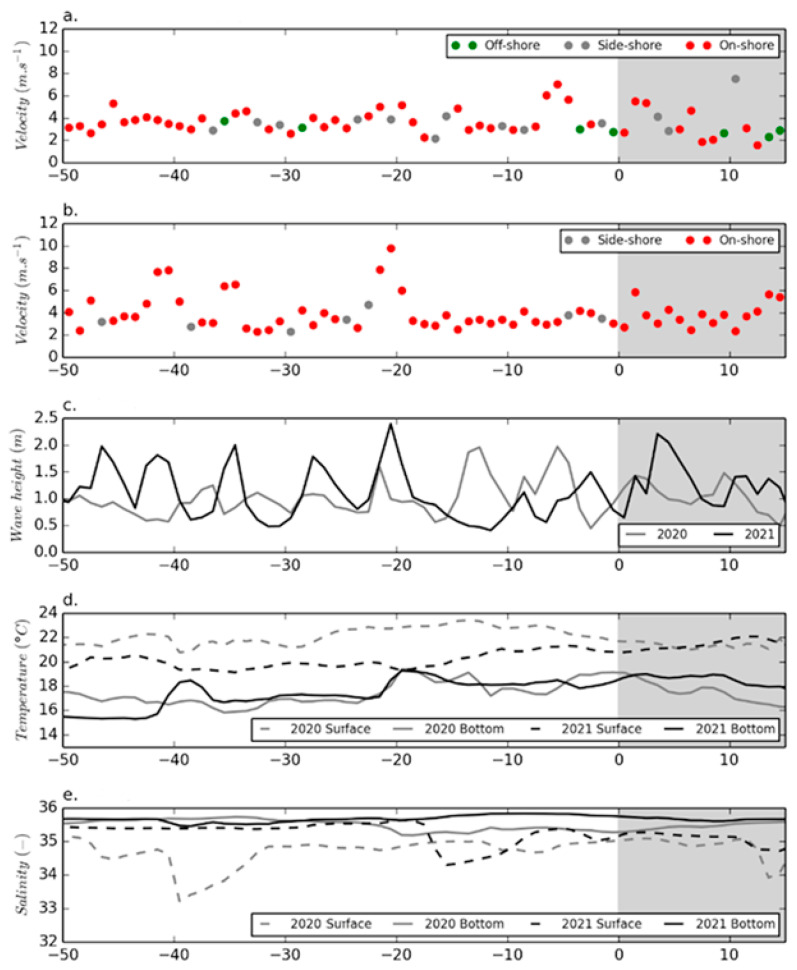
Temporal evolution of meteorological (wind velocity (**a**) in 2020; (**b**) in 2021) and oceanographic (wave height (**c**); water temperature (**d**); and salinity (**e**)) parameters (daily averages) before and at the beginning of both intoxication episodes (shown with gray background). For wind: onshore, coming from 240° to 360°; offshore, coming from 60° to 180°; side-shore, coming from 0° to 60° and 180° to 240°. The *x*-axis shows the number days before and after beginning of the toxic event (with 0 indicating the first intoxication report).

As shown in Figure 4, for both years, the prevailing winds during the weeks preceding the disease reports were onshore. While they were quite strong in the days preceding the 2020 event (end of August), the winds were weak before the 2021 bloom (July). In 2020, the wave height in the 15 days preceding the event was higher than in 2021. In both years, a drop in salinity levels of the surface water was observed from 8 to 15 days before the event. It was more marked in 2021 than in 2020, but bottom water salinities were stable and quite different in both years.

The only common feature of both years in terms of environmental conditions was the sudden rise in the bottom water temperature (above 18 °C) about 20 days before the appearance of symptoms in beach users. Thereafter, this temperature remained fairly high until the beginning of the event in 2020 and for 10 days after the event in 2021 (Figure 4).

### 2.3. Identification of Ostreopsis Species in the Bloom Samples

#### 2.3.1. Cell Morphology in the 2020 Bloom Samples

**Figure 5:**
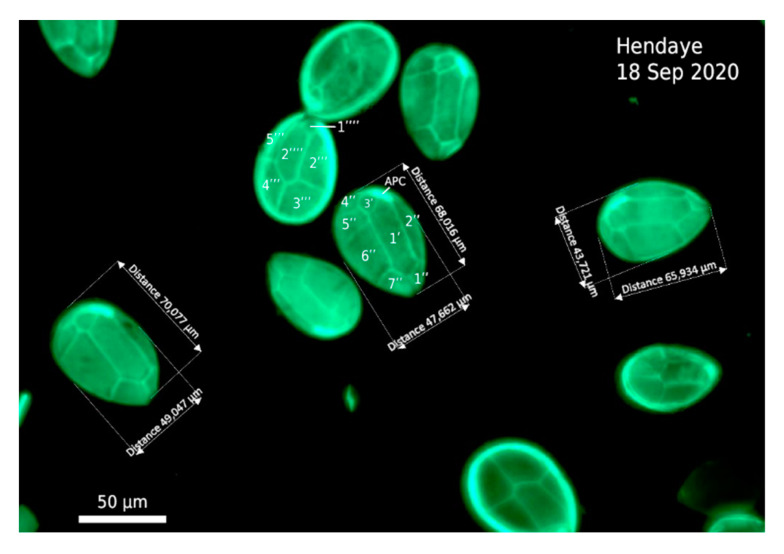
Epiphytic *Ostreopsis* cells from the Hendaye sample (18 September 2020) showing the shape, size, and thecal plate pattern after staining (Solophenyl Flavine 7GFE 500) and observation in epifluorescence microscopy. Scale bar = 50 µm.

During the 2020 bloom event, cells were only observed by light microscopy and by fluorescence to analyze their morphological features and plate pattern. Cells were typically tear-shaped, pointing ventrally, and their size was 55–72 µm deep and 35–50 µm wide, with a continuum of sizes. When observed in epifluorescence microscopy, the thecal plate pattern was typical of the genus, and no difference was seen among cells (Figure 5).

#### 2.3.2. Single Cells Isolated from the 2021 Environmental Sample and Estimation of Relative Abundances of *Ostreopsis* spp.

**Figure 6:**
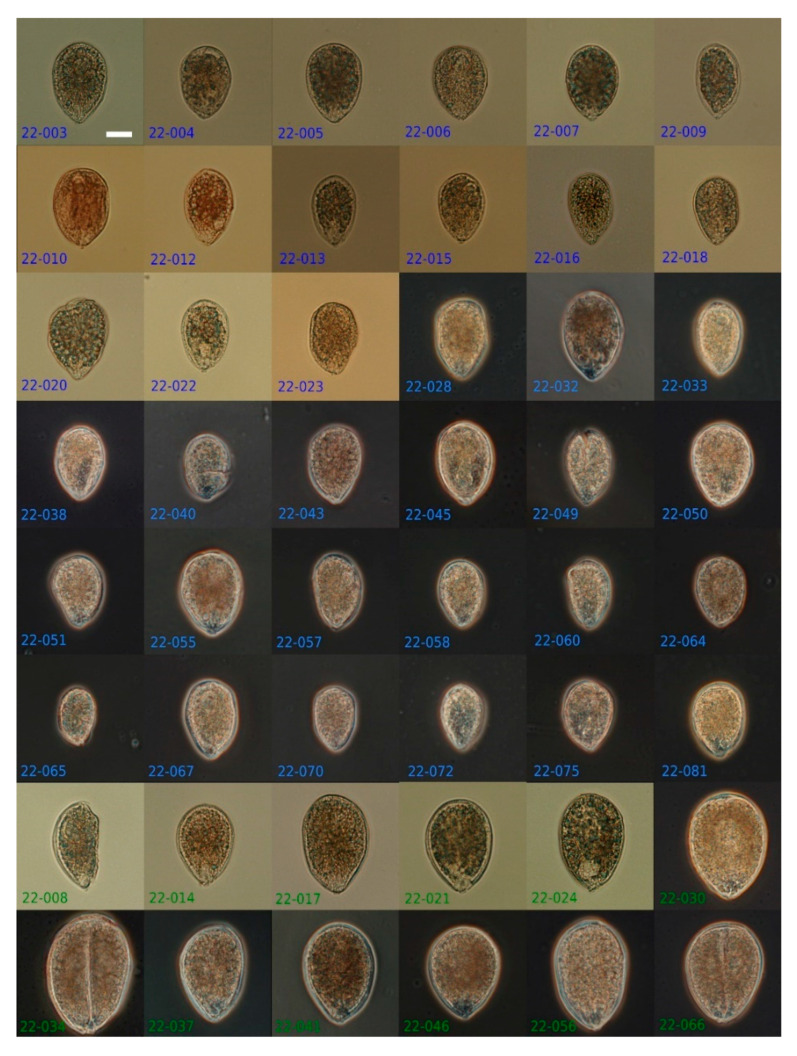
The 48 single *Ostreopsis* cells (on 90) isolated from the Erromardie bloom sample (16 August 2021) for molecular identification. Numbers indicate isolate number, while color corresponds to the obtained genotype: blue = *O.* cf. *ovata*; green = *Ostreopsis* sp. 9. Scale bar = 20 µm.

**Figure 7:**
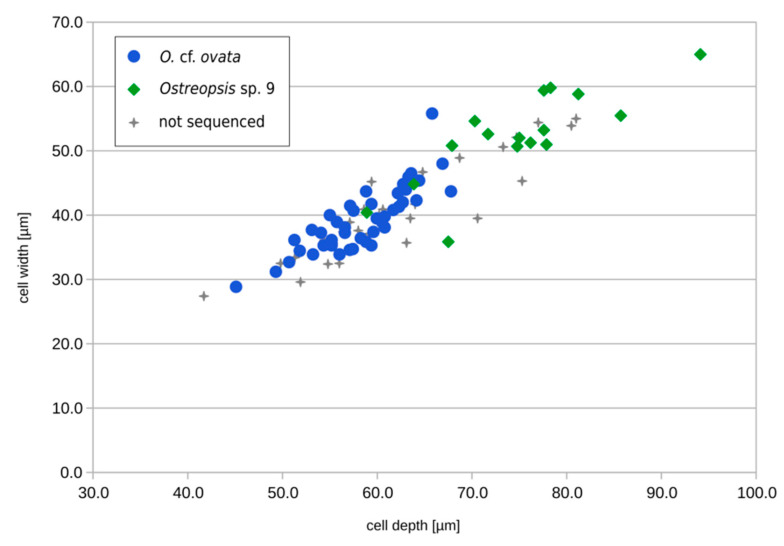
Size distribution of the 90 single cells isolated from the Erromardie environmental sample (16 August 2021) for morpho-molecular identification. Blue dots indicate specimens identified as *O.* cf. *ovata*, and green diamonds indicate specimens identified as *Ostreopsis* sp. 9 by sequencing of the ITS region. Specimens isolated but for which no sequence could be obtained (*n* = 30) are shown as gray crosses.

In order to identify species present in the environmental sample and estimate their relative abundance, 90 *Ostreopsis* cells were isolated randomly. When observed in light microscopy (LM), cells were teardrop-shaped, pointing ventrally ranging from 41.7 to 94.1 µm in depth (dorso-ventral length) and from 27.4 to 65.0 µm in width, with a continuum of sizes (Figure 6 and Figure 7). Except these variations in size, all cells possessed a similar thecal pattern, and no distinctive feature could be observed in LM (Figure 6) or epifluorescence (not shown).

#### 2.3.3. Molecular Phylogeny of Isolated Specimens and Cultivated Strains from the 2021 Bloom

Sequences obtained from both single cells isolated from an environmental sample (*n* = 60) and cultivated strains (*n* = 3) were used in a phylogenetic analysis for species/clade identification. In the maximum-likelihood (ML) tree obtained from the ITS region, 44 environmental sequences and one strain (IFR-OST-01E) grouped with ribotype 1 within subclade A of *O.* cf. *ovata*, while 16 environmental sequences and two strains (IFR-OST-02E, IFR-OST-03E) grouped with ribotype 22 within *Ostreopsis* sp. 9 (*O.* cf. *siamensis*) clade, indicating the co-occurrence of both species in the bloom (Figure 7).

**Figure 8:**
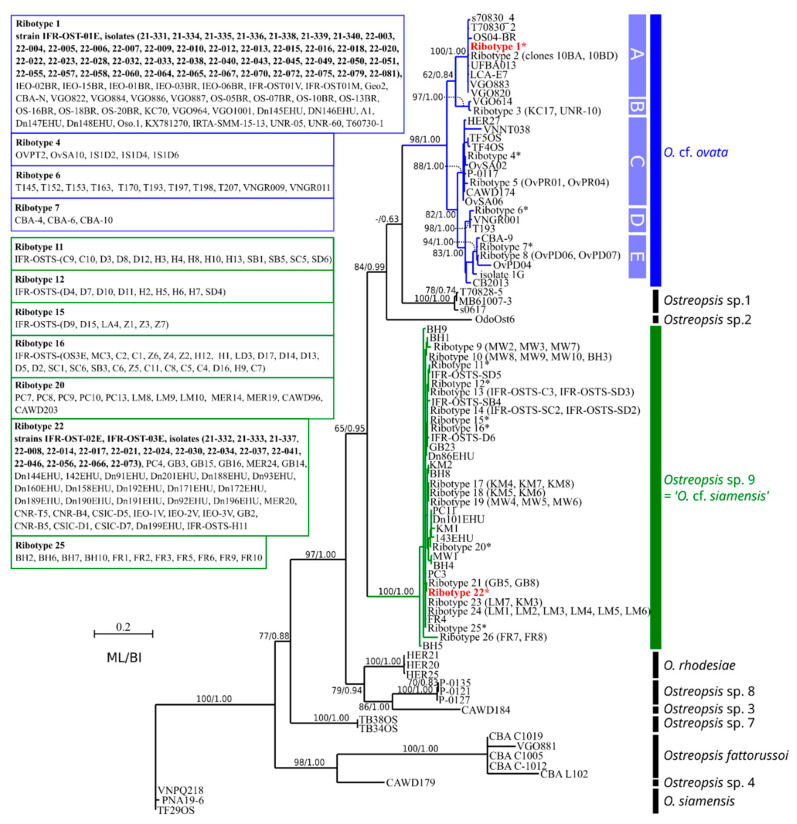
Maximum-likelihood phylogenetic tree inferred from ITS region sequences of various *Ostreopsis* strains and field specimens (88 sequences, 693 sites). Identical sequences are considered to belong to a single ribotype (the list of all sequences used in the analysis is given in Supplementary Table S1). * indicates ribotypes for which sequences are listed in boxes on the left. A–E correspond to subclades in *Ostreopsis* cf. *ovata* clade. Sequences acquired in this study are in bold face. Numbers at nodes indicate bootstrap values (in ML analysis) and posterior probabilities (Bayesian inference). Bootstrap values below 65 are indicated by ‘-’.

As shown by the analysis (Table S1, Figure 8), the subclade A of *O.* cf. *ovata* includes sequences from various origins such as the Mediterranean Sea, Japan, Brazil (southwestern Atlantic), Canary Islands, and southern Portugal (Algarve, eastern Atlantic). Other subclades of *O.* cf. *ovata* included sequences from Australia, Asia (Viet Nam, Thailand, and Malaysia), Ecuador, and Belize. By contrast, all sequences of *Ostreopsis* sp. 9 showed a low level of divergence (Figure 8) although they originated from widely distant areas such as Australia, the Mediterranean Sea, Portugal, and the Basque coast (Atlantic coast of Spain and France).

### 2.4. Toxin Profiles of Ostreopsis Samples

Toxin profiles were determined from environmental samples during the 2021 bloom of *Ostreopsis* spp., as well as from extracts of the three cultivated strains of *Ostreopsis* spp. isolated during the same bloom. Twenty known metabolites produced by *Ostreopsis* spp. were targeted (Table S2) by LC–MS/MS analysis: (i) palytoxin group (PLTX, isob-PLTX, 42-OH-PLTX); (ii) ovatoxin group (OVTX-a to -k); (iii) ostreocins (OST-a, -b, -d, -e); (iv) mascarenotoxin group (McTX-a, -b, -c). Only the PLTX standard is commercially available; the other molecules were, therefore, sought in MRM (mode multiple reaction monitoring) acquisition (i.e., targeted quantitative analysis) on the basis of transitions of published or deduced molecular masses (Table S2).

#### 2.4.1. Toxins Detected in the 2021 Environmental Sample from Erromardie (16 August 2021)

**Figure 9:**
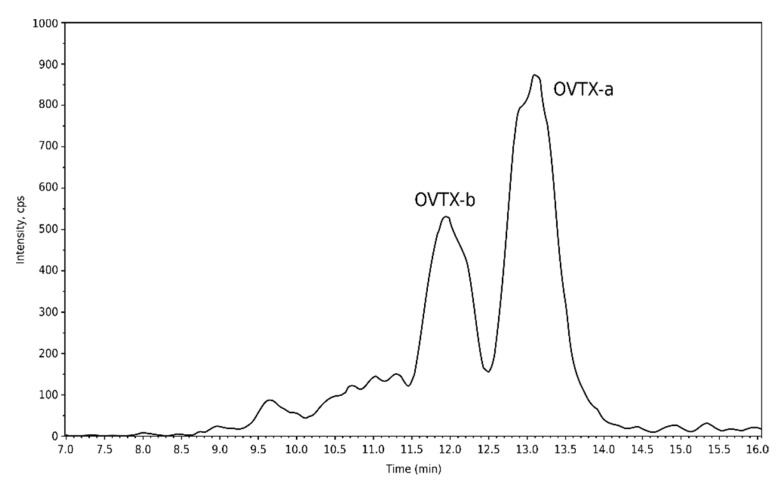
Chromatogram of LC–MS/MS analysis of the sampled bloom from Erromardie (16 August 2021).

Among all metabolites targeted in the study, only two molecules, OVTX-a and -b were quantified in the analyzed environmental sample (Figure 9, Table 1). The intracellular concentration was calculated on the basis of the total abundance of *Ostreopsis* cells in the water (Figure 9, Table 1).

#### 2.4.2. Toxin Profiles of Cultivated Strains

**Figure 10:**
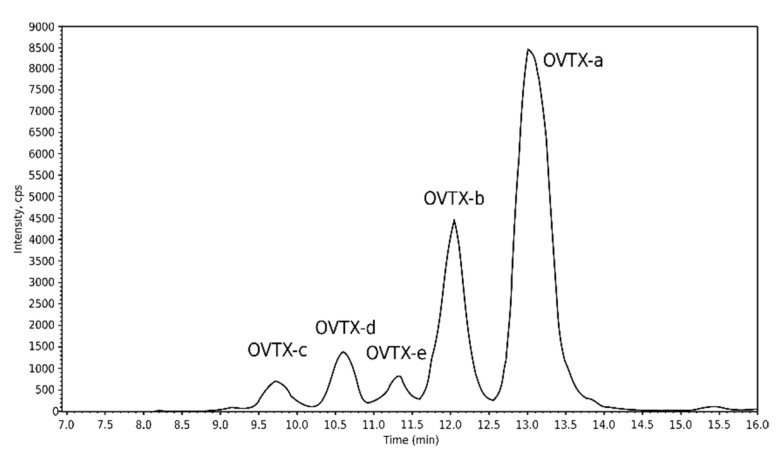
Chromatogram from LC–MS/MS analysis of the strain IFR-OST-01E (*O.* cf. *ovata*) isolated from Erromardie sample.

Culture extracts from the three *Ostreopsis* strains were analyzed, and, among the targeted toxins, only the ovatoxins (OVTX-a, -b, -c, -d, -e) were quantified in strain IFR-OST-01E identified as *Ostreopsis* cf. *ovata* (Figure 10, Table 2). The concentrations obtained are indicated in PLTX equivalents. No targeted toxin was detected in extracts of the two other strains (IFR-OST-02E and IFR-OST-03) identified as *Ostreopsis* sp. 9 (*O.* cf. *siamensis*) (Table 2).

## 3. Discussion

### 3.1. Ostreopsis Bloom Events and Impact on Human Health

As shown by our data, massive respiratory symptoms reported in early August 2021 were concomitant with maximal abundances of *Ostreopsis* spp. observed in the water column and on macroalgae, allowing the health disorders to be associated with the bloom event. The high number of 674 analyzed cases of illness indicates a very important phenomenon on beaches of the Basque coast, and it cannot be excluded that it affected even more people who did not report their symptoms to medical services. In light of this exceptional event, the scarce reports from 2020 could putatively be linked to an *Ostreopsis* outbreak in September; however, due to its late occurrence and the low number of reports, it was not further investigated nor drove specific sampling for molecular and toxins analyses. Hence, a direct link remains hypothetical.

The symptoms reported from the French Basque coast in 2021 (and, to a lesser extent, in 2020) are in perfect agreement with the clinical picture reported in Mediterranean areas [5]. According to the literature, it is probable that PLTX-like toxins (OVTXs) could be at the origin of the observed symptoms of respiratory disorders [51]. Indeed, this type of symptom was already observed in people exposed to aerosols from aquariums containing *Palythoa*, where it is used as a decorative element [53,72]. The reported cases of exposure to PLTX in this context are increasing in Europe and the United States [52,73]. The presence of PLTX-like OVTX-a and isob-PLTX (putative) has been shown in aerosols during an *Ostreopsis* bloom but at a very low concentration (2.4 pg of ovatoxins per liter of air) [27]. Other authors have suggested that fragments of *Ostreopsis*, mucus and/or associated microorganisms (e.g., bacteria) could be present in the aerosol and cause respiratory disorders [56,74].

To date, only blooms of *O.* cf. *ovata* producing OVTXs have been associated with such symptoms occurring in humans, and other species have not caused such health issues although they can form blooms and reach high abundances. For instance, it is now established that the tropical toxic species *O. siamensis* can form benthic blooms and produce ostreocins [18], but effects of these molecules are yet unknown, and their toxicity has only been demonstrated by mouse and neuro-2a cell bioassays [18,43].

### 3.2. Environmental Conditions Associated with the 2020 and 2021 Bloom Events

In 2021, reports of symptoms occurred about 1 month earlier (early August) than in 2020 (in September). Moreover, the detection of the 2021 bloom was primarily initiated by first reports of health issues, and it probably started even earlier in July. Unfortunately, no abundance data of *Ostreopsis* spp. are available for the initiation phase of the bloom, and the complete dynamics remains unknown. Only a decrease in abundances in water (planktonic cells) and on macroalgae (epiphytic cells) was observed from the beginning of August onward. Analysis of environmental parameters before and during the bloom events of both 2020 and 2021 did not allow identifying a specific pattern common for both years, except a rather high seawater temperature (surface exceeding 20 °C and bottom exceeding 18 °C) for a long period before the bloom. Because *Ostreopsis* cells grow on macroalgae attached to rocky bottoms in shallow waters, temperature is generally identified as determining seasonal trends and abundances of this genus [75,76]. In the north of Spain and Basque country, at least three consecutive months with a sea surface temperature exceeding 19.5 °C were identified as a putative trigger of *Ostreopsis* development [77]. Hence, this parameter could be significant, but it needs verification by longer time series in subsequent years.

### 3.3. Identification of Ostreopsis Species during the 2021 Toxic Event

Observed by light microscopy, samples from the blooms of 2020 and 2021 were found to contain abundant *Ostreopsis* cells, but a continuum in body sizes and similar thecal plate pattern did not allow conspicuously identifying different morphotypes. Consequently, species present in the 2020 sample remained unidentified. Due to poor morphological features to distinguish species and the overlap of size ranges, a reliable identification at species level is only possible using molecular methods [14,15,16,21], which was possible only with the 2021 sample. As shown by our results combining morphometric and genetic data, while larger specimens can be attributed to *Ostreopsis* sp. 9 (*O.* cf. *siamensis*) and smaller ones to *O.* cf. *ovata*, the majority of cells in the medium range (depth ca. 60–70 µm) could not be identified solely by light microscopy and required a molecular analysis.

According to sequences acquired from single cells and isolated strains, our study demonstrated the presence of two *Ostreopsis* species in the Erromardie bloom on the French Basque coast. As shown by the phylogenetic analysis, sequences of *Ostreopsis* sp. 9 from the bloom belonged to ribotype 22, which includes other strains previously isolated from Saint-Jean-de-Luz (another site on this French Basque coast), such as Dn201EHU, Dn171EHU, Dn172EHU, and several others originating from the Spanish Basque coast [67] (Table S1), not far from Erromardie beach. Hence, the presence of cells of this ribotype in the 2021 bloom sample is not surprising, since it has been reported in the area for several years [66,67,70]. In a more recent study, a real-time polymerase chain reaction (RT-PCR) approach on many sampling sites confirmed previous observations, and this species was found in several sites between Comillas (Spain) and Biarritz (France) [71]. Furthermore, although no cells could be observed to the north of Biarritz, results from RT-PCR showed that eDNA of *Ostreopsis* sp. 9 has a wider distribution area in the Bay of Biscay, since it could be detected in other sites in the median part of the Bay (Aquitaine) and up to Brittany [71]. Interestingly, no other *Ostreopsis* species have been detected in a previous study in north Spain and Basque coasts, and *Ostreopsis* sp. 9 (*O.* cf. *siamensis*) was considered to be the unique *Ostreopsis* species present in this part of Bay of Biscay [70] until the present work.

It is notable that, although the name *O.* cf. *siamensis* has been widely used for many years for this genotype, it has recently been shown that it differs widely from *O. siamensis*, the type species of the genus, found in tropical areas [18,19]. Since the two organisms are clearly separated, in order to avoid further confusions [2], it has been suggested to use a different name for this genotype; in the present study, we implement the use of a numbered genotype label introduced recently [19].

Interestingly, our genetic analysis demonstrates unambiguously that cells of another genotype, *O.* cf. *ovata* (subclade A), were co-occurring in the 2021 bloom sample. This is, to our knowledge, the first evidence of the presence of this species on the Basque coast and in the Bay of Biscay. In a recent study based on eDNA and RT-PCR methods, where numerous samples collected in summer 2018 on the Spanish and French Basque coast were analyzed, no positive result was obtained for *O.* cf. *ovata* [71]. Given the sensitivity of the method, this indicates that, if present, this species was below the detection limit, and it is probably rare. In light of these data, the hypothesis of a recent introduction and installation of *O.* cf. *ovata* in this part of the Bay of Biscay cannot then be rejected, since, before 2020, *Ostreopsis* proliferations were not associated with human poisonings (Regional Health Agency, pers. com.).

The present data allow extending the distribution area of *O.* cf. *ovata* to the Atlantic Ocean, since, prior to this study, *O.* cf. *ovata* was not reported north of Algarve, in the south of Portugal [67,68,69], for the eastern Atlantic. Hence, the present study allows extending the northern distribution limit of this species in the northeastern Atlantic from the southwestern coast of the Iberian Peninsula (Algarve) to the Cantabrian Sea in the Bay of Biscay (French Basque coast).

The co-occurrence of two species in the Erromardie bloom sample is in agreement with previous findings; *O.* cf. *ovata* (subclade A) was found to co-occur with *Ostreopsis* sp. 9 (e.g., in the Mediterranean Sea, Brazil [14,59], as well as in the Azores and south Portugal [62,67]). From an ecological point of view, the latitude of Erromardie beach (Basque coast) (43.4 ° N) is comparable with some of the Mediterranean areas where *O*. cf. *ovata* was found to form massive blooms, such as Genoa on the Ligurian coast or the Adriatic coast [14,29]. However, the conditions in the Atlantic Ocean are drastically different from those in the Mediterranean Sea, mostly because of a strong tidal effect and different macroalgal communities in both areas. However, there are several reports of blooming *O.* cf. *ovata* in warm regions of the Brazilian Atlantic coast, where the tidal effect is also present, which suggests that temperature might be a more important factor explaining the distribution of this species [60,61].

Further ecological studies aiming at understanding the population dynamics and ecological preferences of both *Ostreopsis* species in the Basque coast should address this question in the future, as it is important for bloom and risk anticipation. Indeed, experimental work in the field and in the laboratory must be carried out to study the dynamics of bloom development and the factors that influence the growth, toxin production, and spatiotemporal distribution of both species on the Basque coast (e.g., temperature, hydrodynamics, nutrients, type of substrate, and salinity).

### 3.4. Toxin Production by Ostreopsis Species during the 2021 Toxic Event

The presence of OVTXs in both environmental and cultivated samples corroborated the identification of *O.* cf. *ovata* as the producer, since these molecules have not been detected for *Ostreopsis* sp. 9. Our analysis on isolated strains confirmed that OVTX was only present in strain IFR-OST01E of *O.* cf. *ovata* and absent in the other strains of *Ostreopsis* sp. 9. The monoclonal culture IFR-OST-01E of *O.* cf. *ovata* contained higher concentrations (on the order of pg·cell^−1^ vs. fg·cell^−1^ for the environmental sample), as well as a more complete toxin profile (OVTX-a to -e vs. OVTX-a and -b for the environmental sample). This particular toxin profile, with a dominance of OVTX-a followed by OVTX-b and a minor presence of OVTX-c and -d, has already been identified in strains of *O.* cf. *ovata* from the Mediterranean Sea and Brazil [39,60,61,78], all genetically belonging to subclade A. The analysis of the toxin profile of 55 strains of *O.* cf. *ovata* collected in the Mediterranean Sea indicates qualitative variability and intraspecific quantitative toxin content [37]. Except for one unique strain, all strains produced OVTXs: 67% strains contained OVTX-a through -e, OVTX-g, and isobaric PLTX; 25% contained OVTX-a, -d, and -e, and isobaric PLTX; 4% produced only OVTX-b and -c; a single compound profile contained OVTX-a to f, with a dominance of OVTX-f, and isobaric PLTX. The OVTX-l analogue was detected in 36 strains [37]. Compared with reports from other areas, the estimated toxin content observed in strain IFR-OST01E (6.7 pg·cell^−1^) is within the same order of magnitude as strains from the Mediterranean Sea reported with OVTXs mostly between 4 and 70 pg·cell^−1^, up to 238 pg·cell^−1^ [37]. However, the estimated concentration is low in comparison to some Brazilian *O.* cf. *ovata* strains containing 31–468 pg·cell^−1^ [61,79] and high in comparison to some strains from the Pacific [41,42]. Comparatively, the apparently low intracellular toxin content observed in the environmental sample (7.8 fg·cell^−1^) can be explained by the presence of both toxic *O.* cf. *ovata* and nontoxic *Ostreopsis* sp. 9, impossible to distinguish in cell counts by LM. According to sequences obtained from single-cells randomly isolated in the bloom sample, *O.* cf. *ovata* appeared to be relatively more abundant (72%) than *Ostreopsis* sp. 9 (28%) in the sample collected 16 August 2021. Since no samples allowing further molecular analysis and toxin analysis were collected earlier, the temporal dynamics of both species and the toxin content during the bloom could not be analyzed. This would be important in future studies since it has been shown that toxin content is dependent on the bloom phase [78].

As previously reported, for instance, for the Mediterranean (Italy) and Atlantic (Portugal) strains of *Ostreopsis* sp. 9 [80], none of the targeted toxins analyzed in this study could be identified in both strains of *Ostreopsis* 9 isolated from the Basque coast. Indeed, it has been suggested that this species presents a much lower risk to human health than *O.* cf. *ovata*, which produces comparatively high levels of PLTX-like compounds [80]. On the basis of cellular assays, a study reported that crude extracts of *Ostreopsis* sp. 9 from several locations along the Andalusian coast contained highly active PLTX-like toxins and may be considered as a possible risk to humans [81], but no compound could be identified. Bioassays on *Artemia franciscana* crustaceans revealed allelopathic effects caused by *Ostreopsis* sp. 9 isolated on the Basque coast, despite the absence of detection of known toxins [82]. In addition, some strains of *Ostreopsis* sp. 9 from southeast Australia were found to produce other than PLTX-like analogues at a very low level (about 0.17 pg·cell^−1^), and they had toxic effects on mice intraperitoneally with a very high dose of extract (LD_50_ of 25 mg·kg^−1^) of the studied strain, versus only 0.15 to 0.72 μg·kg^−1^ for PLTX [83]. Hence, it is highly probable that nonidentified toxic compounds in this species cause negative effects on marine benthic organisms, but this is considerably less studied than for *O.* cf. *ovata* [84] and further work is necessary.

## 4. Materials and Methods

### 4.1. Sampling on the Basque Coast

In 2020, one macroalgal sample (*Ericaria selaginoides*) was collected at Hendaye on 18 September (Figure 1, Table S3). In 2021, after first reports of symptoms (2 August 2021), water and macroalgae were collected at Erromardie beach (Figure 1), and, since the phenomenon was extending to other areas, other samples were collected at two other beaches (Viviers Basque, Parlementia) during the following weeks (Figure 1). Table S3 summarizes the samples collected for the different analyses realized in the study.

Water samples were collected using 500 mL plastic flasks sunk into the water at a depth of about 30 cm in the vicinity of attached macroalgae. Macroalgal samples were collected with surrounding water at a depth of about 30 cm, using 250 mL plastic flasks. After shaking the bottle for about 5 min, the macroalgae were removed and drained, and their fresh weight (FW) was measured. Epiphytic cells collected were fixed with acidic Lugol’s solution (2% *v*/*v*) for abundance estimations of *Ostreopsis* cells.

Water samples for cell counts were fixed with acidic Lugol’s solution (2% *v*/*v*) and stored in the dark. For molecular identification of *Ostreopsis* cells, the sample was fixed with 95% ethanol (final concentration) and stored at 4 °C. For isolating strains, samples were kept fresh with no direct exposure to light.

For toxin analysis of water samples, after counting the cells, 60 L of seawater was filtered through a 47 mm GF/F filter (Whatman) (Erromardie, 16 August 2021, Table S3) in duplicate, and the two filters were kept frozen at −20 °C until analysis.

### 4.2. Cell Counts and Estimation of Ostreopsis Abundances

Cells from the Lugol-fixed samples were observed using a Zeiss Axio Observer microscope (Carl Zeiss, Oberkochen, Germany) equipped with a digital camera. Counts were realized in 10 mL settling chambers at ×100 magnification and using phase-contrast optics. For visualization of the thecal plates, cells were stained with Solophenyl Flavine 7GFE 500 and observed in epifluorescence microscopy with the same microscope equipped with a GFP filterset [85].

### 4.3. Environmental Parameters

Environmental parameters were obtained from different meteorological and oceanographic models; wind speed and direction were results from the AROME regional model (Meteo France forecasting system, spatial resolution of 2500 m), wave height was from WaveWatchIII simulations for the NORGAS_UG configuration (spatial resolution of 500 m), and water temperature and salinity were from MARS3D simulations for the MANGAE_2500 configuration (spatial resolution of 2500 m) [86].

### 4.4. Strains Isolation and Cultivation

Cells were isolated with a micropipette using an inverted IM35 microscope (Carl Zeiss, Oberkochen, Germany), rinsed in several drops of filtered seawater, and put in the well of a four-well microplate (Thermo Scientific^®^, Waltham, MA, United States). Three monoclonal strains of *Ostreopsis* spp. were grown in L1 culture medium [87] prepared with filtered seawater at pH 8.2 and a salinity of 35. They were maintained in 250 mL Erlenmeyer flasks placed in a thermoregulated room at 17 °C and under 160 µmol·quanta m^2^·s^−1^ PAR (photosynthetically active radiation) on a 16 h light/8 h dark cycle. Irradiance was delivered by Osram Fluora 36W (Munich, Germany) and Philips Daylight 36W (Amsterdam, the Netherlands) lamps.

### 4.5. Species Identification and Estimation of Their Relative Abundances

To identify species present during the bloom, sequencing of the ITS1-5.8S rDNA-ITS2 (ITS region) was realized on single cells isolated both from the ethanol-fixed environmental sample and from strains in culture.

#### 4.5.1. Single-Cells Isolation

For identification and a rough estimation of the relative abundance of *Ostreopsis* spp. In the ethanol-fixed bloom sample (16 August 2021), 90 single cells were isolated randomly from a 5 mL subsample settled in a sedimentation chamber using an IX51 (Olympus, Tokyo, Japan) inverted microscope. Cells were isolated with a glass micropipette, transferred to a microscope slide, photographed to record their overall morphology, rinsed in several drops of ultrapure deionized water, and then transferred into a 0.2 mL PCR tube for molecular analysis.

#### 4.5.2. DNA Extraction from Strains

The three live cultures (IFR-OST-01E, IFR-OST-02E, and IFR-OST-03E) were extracted with the PCRBIO Rapid Extract PCR Kit (PCR Biosystems Ltd., London, United Kingdom) which combines extraction and PCR. A volume of 70 µL of culture was transferred into a 0.5 mL tube and centrifuged for 3 min at 14,000× *g*. The supernatant was discarded to retain only the pellet. Then, the manufacturer’s instructions were followed except for the dilution step where 190 µL of water was added instead of the recommended 900 µL. The pair of primers used for the PCR was ITS-FW (GTAGGTGAACCTGCGGAAGG) and D3B (TCGGAGGGAACCAGCTACTA) [88,89].

#### 4.5.3. Amplification and Sequencing

For PCR, the tubes were processed as described previously [18] using Promega Master Mix (Promega, France) according to the manufacturer’s instructions. Thermal cycling conditions comprised an initial 2 min heating step at 95 °C, followed by 35 cycles of 95 °C for 30 s, 60 °C for 60 s, 72 °C for 120 s, and a final extension at 72 °C for 5 min. Positive samples revealed by visualization on an agarose gel (2%) after electrophoresis were selected for sequencing. A purification using the ExoSAP-IT Product Cleanup reagent (Affymetrix, Cleveland, OH, USA) was realized prior to the sequencing reaction with Big Dye X-terminator v. 3.1 Sequencing Kit (Applied Biosystems, Foster City, CA, USA). Excess labeled nucleotides were removed using a Bid Dye X-terminator purification kit, and sequencing products were run on an ABI PRISM 3130 Genetic Analyzer. Forward and reverse reads were obtained.

#### 4.5.4. Phylogenetic Analysis

Prior to phylogenetic analysis inferred from the ITS1-5.8S rDNA-ITS2 region, *Ostreopsis* sequences related to those obtained in the study were identified using the BLAST tool of NCBI. In addition to the 48 sequences acquired, a total of 267 sequences were retrieved from GenBank (Table S1) including 82 sequences for *O.* cf. *ovata* and 163 sequences for *Ostreopsis* sp. 9 (*O.* cf. *siamensis*; Table S1). In order to limit the number of repeated sequences in the analysis, when multiple clones share a similar sequence, they were considered as a single ribotype. Hence, the dataset used for phylogenetic analysis included 88 sequences. Sequences were aligned using MAFFT v. 7 with the q-ins-i option [90] and manual refinement. Maximum-likelihood analysis was performed using PHY-ML v. 3.0 software [91], and a bootstrap analysis (1000 pseudoreplicates) was used to assess the relative robustness of branches of the ML tree. In addition, a Bayesian inference (BI) analysis was run using MrBayes v. 3.1.2 [92].

### 4.6. Toxin Analysis by Liquid Chromatography Tandem Mass Spectrometry (LC–MS/MS)

Samples were screened for the presence of PLTX and related analogues (20 analogues; see Table S2).

#### 4.6.1. Sample Preparation

- Environmental sample

In order to extract environmental *Ostreopsis* cells collected on the filters, the two GF/F filters were thawed. Then, 3 mL MeOH was added to each filter, and the filters were ultrasonicated in an ultrasonic bath at 25 Hz for 5 min. Then, after centrifugation (4300× *g* for 5 min at 6 °C), the resulting pellet was extracted again with 2 mL of MeOH, both supernatants were pooled, and the volume was adjusted to 5 mL, before analysis by LC–MS/MS.

- Strains in culture

Pellets of the three *Ostreopsis* strains (containing approximately 85,000 cells·mL^−1^), were extracted with 500 µL of MeOH using an ultrasonic bath at 25 Hz for 10 min. After centrifugation (4300× *g* for 5 min at 6 °C), the resulting pellet was extracted twice with 500 µL MeOH, both supernatants were pooled, and the volume was adjusted to 1 mL. The supernatants were then analyzed by LC–MS/MS.

#### 4.6.2. LC–MS/MS Analysis

The supernatants were ultrafiltered (0.20 μm, Nanosep MF, Pall, Mexico) before LC–MS/MS analyses. Liquid chromatography was performed on a Poroshell 120 EC-C18 column (100 × 2.1 mm, 2.7 μm, Agilent, Les Ulis, France) equipped with a guard column (5 × 2.1 mm, 2.7 μm, same stationary phase) using a Nexera Ultra-Fast Liquid Chromatography system (Prominence UFLC-XR, Shimadzu, France). Gradients of water (A) and acetonitrile 95% (B) both containing 0.2% acetic acid were used at a flow rate of 0.2 mL·min^−1^. The injection volume was 5 μL, and the column temperature was 25 °C. MS/MS analyses were performed with an API 4000QTRAP (AB Sciex, Les Ulis, France) in positive ion mode and using MRM (Multiple Reaction Monitoring) acquisition. UV detection at 220, 233, 263, and 220–360 nm was performed with a diode array detector (Prominence, SPD-M20A, Shimadzu, France).

Lastly, methods described in [16] were used to detect PLTX, 42-OH-PLTX, 12 OVTXs (-a to -k), four OSTs (-A, -B, -D, and -E1), and three McTXs (A to C), corresponding to 20 compounds [16]. The used precursors and product ions used, as well as wavelengths for detection of these compounds, were similar to those used in [16]. Quantification was performed relative to the PLTX standard (Wako Chemicals GmbH, Germany) using a nine-point calibration curve. The limits of detection and quantification were 20 and 30 ng·mL^−1^ for the PLTX standard.

## 5. Conclusions

As revealed by our analysis, the toxic *Ostreopsis* bloom, which occurred in summer 2021 on the French Basque coast and affected about 700 people, was not monospecific and included two species: *Ostreopsis* sp. 9 (*O.* cf. *siamensis*) and *O.* cf. *ovata*. From this result, it can be suspected that a similar bloom caused the health disorders reported in September 2020, but the low number of cases did not match the massive issues in 2021. While the former is a well-known species reported in the area for several years but usually with no noticeable effects on the population, the latter was found for the first time in this part of Bay of Biscay. Interestingly, *O.* cf. *ovata* was not previously detected in this area despite a sampling in 2018 at a large geographical scale on the French Atlantic coast and the use of sensitive molecular methods in a previous study in 2018 [71]. Hence, the hypothesis of a recent introduction cannot be excluded, and the presence of this invasive dinoflagellate might cause new problems in the future, in relation to global warming. This problematic species, well established in the Mediterranean Sea, as well as on the southwestern Atlantic (Brazilian coasts) extends its distribution area in the northern Atlantic. The toxin profile with OVTX-a and -b as predominant compounds in the environmental sample corroborates the presence of *O.* cf. *ovata*, since these molecules have only been found in this species, and they were likely produced by this taxon in the bloom, as revealed by analysis of clonal strains. Furthermore, it would be important to analyze the cytotoxic effects of both species on human and marine fauna using bioassays. Such approaches are required to better evaluate the toxic risk associated with *Ostreopsis* blooms on the Basque coast.

## Figures and Tables

**Table 1:** Toxins quantified in the environmental bloom sample from Erromardie (16 August 2021). The concentrations are indicated in PLTX equivalents.

Sample Analyzed	Toxins	Cellular Content (fg eq. PLTX·Cell^−1^)
Bloom water sample (16 August 2021)	OVTX-a	5.5 ^1^
	OVTX-b	2.3 ^1^

^1^ Based on the total abundance of *Ostreopsis* cells in the sample.

**Table 2:** Toxins quantified in cultured strains of *Ostreopsis* from Erromardie bloom. The concentrations are indicated in PLTX equivalents.

Strain	Species	Concentrations (pg eq. PLTX·Cell^−1^)					Cell Concentration (Cells·mL^−1^)
IFR-OST-01E	*Ostreopsis* cf. *ovata*	OVTX-a	OVTX-b	OVTX-c	OVTX-d	OVTX-e	80,700
		4.3	1.6	0.2	0.3	0.3	
IFR-OST-02E	*Ostreopsis* sp. 9 (*O*. cf. *siamensis*)	No targeted toxins detected					96,300
IFR-OST-03E	*Ostreopsis* sp. 9 (*O.* cf. *siamensis*)	No targeted toxins detected					84,000

## Data Availability

Not applicable.

## Supplementary Materials

The following supporting information can be downloaded at https://www.mdpi.com/article/10.3390/md20070461/s1: Table S1, List of strains and sequences used in the phylogenetic analysis; Table S2, Molecular ion transitions (Q1 > Q3) and wavelengths used for the detection of palytoxin and its analogues (ovatoxins, ostreocins, and mascarenotoxins) [31,33,34,35,36,44,46,47,80,93,94,95,96,97]; Table S3, Summary of samples collected for the different analyses.
