# Supplementary material for: First Characterization of Ostreopsis cf. ovata (Dinophyceae) and Detection of Ovatoxins during a Multispecific and Toxic Ostreopsis Bloom on French Atlantic Coast
Source: Mar Drugs. 2022 Jul 18;20(7):461. doi: 10.3390/md20070461 (PMC9315632; doi:10.3390/md20070461)
Supplement: Supplementary file 1 [file marinedrugs-20-00461-s001.zip › Table S1.pdf]

Table S1. List of strains/sequences used in the phylogenetic analysis. Clones sharing a similar sequence are considered as the same ribotype.

Strain / isolate ID	ribotype	Accession number	Origin	Clade
S70830-4		AB674907	Japan : Susaki City, Kochi	*O.* cf. *ovata* (subclade A)
T70828-2		AB674903	Japan: Tei, Konan City, Kochi	*O.* cf. *ovata* (subclade A)
OS-04BR		FM244664	Brazil:West Atlantic Ocean, Rio de Janeiro	*O.* cf. *ovata* (subclade A)
T60730-1	1	AB674902	Japan: Konan City, Kochi	*O.* cf. *ovata* (subclade A)
A1	1	AJ311520	Italy:Gioia Tauro,Tyrrhenian Sea	*O.* cf. *ovata* (subclade A)
IEO-02BR	1	AJ318461	Brazil	*O.* cf. *ovata* (subclade A)
IEO-15BR	1	AJ318462	Brazil	*O.* cf. *ovata* (subclade A)
IEO-01BR	1	AJ420006	Brazil	*O.* cf. *ovata* (subclade A)
IEO-03BR	1	AJ491311	Brazil	*O.* cf. *ovata* (subclade A)
IEO-06BR	1	AJ491312	Brazil	*O.* cf. *ovata* (subclade A)
**IFR-OST-01E**	**1**	**ON980637**	**France: Saint-Jean-de-Luz**	*O.* cf. *ovata* (subclade A)
**IFR21-331**	**1**	**ON980577**	**France: Saint-Jean-de-Luz**	*O.* cf. *ovata* (subclade A)
**IFR21-334**	**1**	**ON980578**	**France: Saint-Jean-de-Luz**	*O.* cf. *ovata* (subclade A)
**IFR21-335**	**1**	**ON980579**	**France: Saint-Jean-de-Luz**	*O.* cf. *ovata* (subclade A)
**IFR21-336**	**1**	**ON980580**	**France: Saint-Jean-de-Luz**	*O.* cf. *ovata* (subclade A)
**IFR21-338**	**1**	**ON980581**	**France: Saint-Jean-de-Luz**	*O.* cf. *ovata* (subclade A)
**IFR21-339**	**1**	**ON980582**	**France: Saint-Jean-de-Luz**	*O.* cf. *ovata* (subclade A)
**IFR21-340**	**1**	**ON980583**	**France: Saint-Jean-de-Luz**	*O.* cf. *ovata* (subclade A)
**IFR22-003**	**1**	**ON980584**	**France: Saint-Jean-de-Luz**	*O.* cf. *ovata* (subclade A)
**IFR22-004**	**1**	**ON980585**	**France: Saint-Jean-de-Luz**	*O.* cf. *ovata* (subclade A)
**IFR22-005**	**1**	**ON980586**	**France: Saint-Jean-de-Luz**	*O.* cf. *ovata* (subclade A)
**IFR22-006**	**1**	**ON980587**	**France: Saint-Jean-de-Luz**	*O.* cf. *ovata* (subclade A)
**IFR22-007**	**1**	**ON980588**	**France: Saint-Jean-de-Luz**	*O.* cf. *ovata* (subclade A)
**IFR22-009**	**1**	**ON980589**	**France: Saint-Jean-de-Luz**	*O.* cf. *ovata* (subclade A)
**IFR22-010**	**1**	**ON980590**	**France: Saint-Jean-de-Luz**	*O.* cf. *ovata* (subclade A)
**IFR22-012**	**1**	**ON980591**	**France: Saint-Jean-de-Luz**	*O.* cf. *ovata* (subclade A)
**IFR22-013**	**1**	**ON980592**	**France: Saint-Jean-de-Luz**	*O.* cf. *ovata* (subclade A)
**IFR22-015**	**1**	**ON980593**	**France: Saint-Jean-de-Luz**	*O.* cf. *ovata* (subclade A)
**IFR22-016**	**1**	**ON980594**	**France: Saint-Jean-de-Luz**	*O.* cf. *ovata* (subclade A)
**IFR22-018**	**1**	**ON980595**	**France: Saint-Jean-de-Luz**	*O.* cf. *ovata* (subclade A)
**IFR22-020**	**1**	**ON980596**	**France: Saint-Jean-de-Luz**	*O.* cf. *ovata* (subclade A)
**IFR22-022**	**1**	**ON980597**	**France: Saint-Jean-de-Luz**	*O.* cf. *ovata* (subclade A)
**IFR22-023**	**1**	**ON980598**	**France: Saint-Jean-de-Luz**	*O.* cf. *ovata* (subclade A)
**IFR22-028**	**1**	**ON980599**	**France: Saint-Jean-de-Luz**	*O.* cf. *ovata* (subclade A)
**IFR22-032**	**1**	**ON980600**	**France: Saint-Jean-de-Luz**	*O.* cf. *ovata* (subclade A)
**IFR22-033**	**1**	**ON980601**	**France: Saint-Jean-de-Luz**	*O.* cf. *ovata* (subclade A)
**IFR22-038**	**1**	**ON980602**	**France: Saint-Jean-de-Luz**	*O.* cf. *ovata* (subclade A)
**IFR22-040**	**1**	**ON980603**	**France: Saint-Jean-de-Luz**	*O.* cf. *ovata* (subclade A)
**IFR22-043**	**1**	**ON980604**	**France: Saint-Jean-de-Luz**	*O.* cf. *ovata* (subclade A)
**IFR22-045**	**1**	**ON980605**	**France: Saint-Jean-de-Luz**	*O.* cf. *ovata* (subclade A)
**IFR22-049**	**1**	**ON980606**	**France: Saint-Jean-de-Luz**	*O.* cf. *ovata* (subclade A)
**IFR22-050**	**1**	**ON980607**	**France: Saint-Jean-de-Luz**	*O.* cf. *ovata* (subclade A)
**IFR22-051**	**1**	**ON980608**	**France: Saint-Jean-de-Luz**	*O.* cf. *ovata* (subclade A)
**IFR22-055**	**1**	**ON980609**	**France: Saint-Jean-de-Luz**	*O.* cf. *ovata* (subclade A)
**IFR22-057**	**1**	**ON980610**	**France: Saint-Jean-de-Luz**	*O.* cf. *ovata* (subclade A)
**IFR22-058**	**1**	**ON980611**	**France: Saint-Jean-de-Luz**	*O.* cf. *ovata* (subclade A)
**IFR22-060**	**1**	**ON980612**	**France: Saint-Jean-de-Luz**	*O.* cf. *ovata* (subclade A)
**IFR22-064**	**1**	**ON980613**	**France: Saint-Jean-de-Luz**	*O.* cf. *ovata* (subclade A)
**IFR22-065**	**1**	**ON980614**	**France: Saint-Jean-de-Luz**	*O.* cf. *ovata* (subclade A)
**IFR22-067**	**1**	**ON980615**	**France: Saint-Jean-de-Luz**	*O.* cf. *ovata* (subclade A)
**IFR22-070**	**1**	**ON980616**	**France: Saint-Jean-de-Luz**	*O.* cf. *ovata* (subclade A)

**IFR22-072**	**1**	**ON980617**	**France: Saint-Jean-de-Luz**	*O.* cf. *ovata* (subclade A)
**IFR22-075**	**1**	**ON980618**	**France: Saint-Jean-de-Luz**	*O.* cf. *ovata* (subclade A)
**IFR22-079**	**1**	**ON980619**	**France: Saint-Jean-de-Luz**	*O.* cf. *ovata* (subclade A)
**IFR22-081**	**1**	**ON980620**	**France: Saint-Jean-de-Luz**	*O.* cf. *ovata* (subclade A)
IFR-OST01V	1	FJ905896	France: Villefranche sur Mer	*O.* cf. *ovata* (subclade A)
IFR-OST01M	1	FJ905897	France: Morgiret, Frioul Island, Marseilles	*O.* cf. *ovata* (subclade A)
Geo-2	1	FM244627	Italy:Mediterranean, Ligurian, Genova	*O.* cf. *ovata* (subclade A)
CBA-N	1	FM244631	Italy:Tyrrhenian Sea, S. Felice Circeo	*O.* cf. *ovata* (subclade A)
VGO822	1	FM244635	Spain:Mediterranean, Catalan, Costa Brava	*O.* cf. *ovata* (subclade A)
VGO884	1	FM244639	Spain:East Atlantic, Canary Island, Lanzarote	*O.* cf. *ovata* (subclade A)
VGO886	1	FM244640	Spain:East Atlantic, Canary Island, Lanzarote	*O.* cf. *ovata* (subclade A)
VGO887	1	FM244641	Spain:East Atlantic, Canary Island, Lanzarote	*O.* cf. *ovata* (subclade A)
OS-05BR	1	FM244665	Brazil:West Atlantic Ocean, Rio de Janeiro	*O.* cf. *ovata* (subclade A)
OS-07BR	1	FM244666	Brazil:West Atlantic Ocean, Rio de Janeiro	*O.* cf. *ovata* (subclade A)
OS-10BR	1	FM244667	Brazil:West Atlantic Ocean, Rio de Janeiro	*O.* cf. *ovata* (subclade A)
OS-13BR	1	FM244668	Brazil:West Atlantic Ocean, Rio de Janeiro	*O.* cf. *ovata* (subclade A)
OS-16-BR	1	FM244669	Brazil:West Atlantic Ocean, Rio de Janeiro	*O.* cf. *ovata* (subclade A)
OS-18BR	1	FM244670	Brazil:West Atlantic Ocean, Rio de Janeiro	*O.* cf. *ovata* (subclade A)
OS-20BR	1	FM244671	Brazil:West Atlantic Ocean, Rio de Janeiro	*O.* cf. *ovata* (subclade A)
KC70	1	FM244734	Greece:Mediterranean, Aegean, Athens	*O.* cf. *ovata* (subclade A)
VGO964	1	HG972965	Spain:Llavaneres, Catalan Sea	*O.* cf. *ovata* (subclade A)
VGO10001	1	JX065551	Spain: East Atlantic, Canary Island, Lanzarote	*O.* cf. *ovata* (subclade A)
Dn145EHU	1	JX987684	Portugal: Lagos	*O.* cf. *ovata* (subclade A)
Dn146EHU	1	JX987685	Portugal: Lagos	*O.* cf. *ovata* (subclade A)
Dn147EHU	1	JX987686	Portugal: Lagos	*O.* cf. *ovata* (subclade A)
Dn148EHU	1	JX987687	Portugal: Lagos	*O.* cf. *ovata* (subclade A)
Oso.1	1	KJ781415	Portugal: Lagos	*O.* cf. *ovata* (subclade A)
–	1	KX781270	Brazil: Sao Paulo, Ubatuba	*O.* cf. *ovata* (subclade A)
IRTA-SMM15-13	1	MG551865	Spain: Fangar Bay	*O.* cf. *ovata* (subclade A)
UNR-05	1	MN560106	Brazil: Rio de Janeiro, Armacao dos Buzios, Praia do Forno	*O.* cf. *ovata* (subclade A)
UNR-60	1	MN560108	Brazil: Pernambuco, Fernando de Noronha	*O.* cf. *ovata* (subclade A)
VGO820		FM244634	Spain:Mediterranean, Catalan, Costa Brava	*O.* cf. *ovata* (subclade A)
10BD	2	KF612633	Portugal: Atlantic coast in Camilo, Algarve	*O.* cf. *ovata* (subclade A)
10BA	2	KF612635	Portugal: Atlantic coast in Camilo, Algarve	*O.* cf. *ovata* (subclade A)
UFBA013		KY628450	Brazil	*O.* cf. *ovata* (subclade A)
LCA-E7		JF682767	Brazil	*O.* cf. *ovata* (subclade A)
VGO883		FM244638	Spain:East Atlantic, Canary Island, Lanzarote	*O.* cf. *ovata* (subclade A)
VGO614		FM244642	Portugal:East Atlantic, Madeira, Bahia de Abra	*O.* cf. *ovata* (subclade B)
KC17	3	FM244736	Greece:Mediterranean, N Aegean, Limnos Island	*O.* cf. *ovata* (subclade B)
UNR-10	3	MN560107	Brazil: Rio Grande do Norte, Tibau do Sul, Praia de Pipa	*O.* cf. *ovata* (subclade B)
HER27		KX055881	Australia : Heron Reef (Queensland)	*O.* cf. *ovata* (subclade C)
VNNT038		MT968505	Viet Nam	*O.* cf. *ovata* (subclade C)
TF4OS		AB841250	Thailand:Trat, Koh Wai	*O.* cf. *ovata* (subclade C)
TF5OS		AB841251	Thailand:Trat, Koh Wai	*O.* cf. *ovata* (subclade C)
OVPT2	4	AB674905	Malaysia	*O.* cf. *ovata* (subclade C)
OvSA10	4	AF218464	Malaysia: Kota Kinabalu	*O.* cf. *ovata* (subclade C)
1S1D2	4	KX129869	China	*O.* cf. *ovata* (subclade C)
1S1D4	4	KX129870	China	*O.* cf. *ovata* (subclade C)
1S1D6	4	KX129871	China	*O.* cf. *ovata* (subclade C)

OvSA02		AF218460	Malaysia: Kota Kinabalu	*O.* cf. *ovata* (subclade C)
P-0117		KM032202	France: Reunion Island, W Indian Ocean	*O.* cf. *ovata* (subclade C)
OvPR01	5	AF218457	Malaysia: Pulau Redang	*O.* cf. *ovata* (subclade C)
OvPR04	5	AF218458	Malaysia: Pulau Redang	*O.* cf. *ovata* (subclade C)
CAWD174		AB674904	Japan: Kubazaki, Kohama island	*O.* cf. *ovata* (subclade C)
OvSA06		AF218463	Malaysia: Kota Kinabalu	*O.* cf. *ovata* (subclade C)
T145	6	AB841232	Thailand:Rayong, Koh Man	*O.* cf. *ovata* (subclade D)
T152	6	AB841235	Thailand:Rayong, Koh Man	*O.* cf. *ovata* (subclade D)
T153	6	AB841236	Thailand:Rayong, Koh Man	*O.* cf. *ovata* (subclade D)
T163	6	AB841237	Thailand:Rayong, Koh Man	*O.* cf. *ovata* (subclade D)
T170	6	AB841238	Thailand:Rayong, Koh Man	*O.* cf. *ovata* (subclade D)
T197	6	AB841245	Thailand:Rayong, Koh Man	*O.* cf. *ovata* (subclade D)
T198	6	AB841246	Thailand:Rayong, Koh Man	*O.* cf. *ovata* (subclade D)
T207	6	AB841248	Thailand:Rayong, Koh Man	*O.* cf. *ovata* (subclade D)
VNGR009	6	MT968503	Viet Nam	*O.* cf. *ovata* (subclade D)
VNGR011	6	MT968504	Viet Nam	*O.* cf. *ovata* (subclade D)
VNGR001		MT968502	Viet Nam	*O.* cf. *ovata* (subclade D)
T193		AB841241	Thailand:Rayong, Koh Man	*O.* cf. *ovata* (subclade D)
CBA-9		FM244726	Indonesia:East Pacific, North Sulawesi	*O.* cf. *ovata* (subclade E)
CBA-4	7	FM244724	Indonesia:East Pacific, North Sulawesi	*O.* cf. *ovata* (subclade E)
CBA-6	7	FM244725	Indonesia:East Pacific, North Sulawesi	*O.* cf. *ovata* (subclade E)
CBA-10	7	FM244727	Indonesia:East Pacific, North Sulawesi	*O.* cf. *ovata* (subclade E)
OvPD06	8	AF218455	Malaysia: Port Dickson	*O.* cf. *ovata* (subclade E)
OvPD07	8	AF218456	Malaysia: Port Dickson	*O.* cf. *ovata* (subclade E)
OvPD04		AF076217	Malaysia: Port Dickson	*O.* cf. *ovata* (subclade E)
1G		MH844087	Ecuador: Galapagos	*O.* cf. *ovata* (subclade E)
CB-2013		JX065586	Belize: Caribbean Sea	*O.* cf. *ovata* (subclade E)
T70828-5		AB674908	Japan: Tei, Konan City, Kochi	*Ostreopsis* sp. 1
MB61007-3		AB674909	Japan: Bishagoiwa, Muroto City, Kochi	*Ostreopsis* sp. 1
s0617		AB674910	Japan: Misaki cho, Nagasaki city, Nagasaki	*Ostreopsis* sp. 1
OdoOst6		AB674913	Japan: Odo, Itoman, Okinawa	*Ostreopsis* sp. 2
BH9		MN418266	Australia	*Ostreopsis* sp. 9
BH1		MN418258	Australia	*Ostreopsis* sp. 9
MW2	9	MN418249	Australia	*Ostreopsis* sp. 9
MW3	9	MN418250	Australia	*Ostreopsis* sp. 9
MW7	9	MN418254	Australia	*Ostreopsis* sp. 9
MW8	10	MN418255	Australia	*Ostreopsis* sp. 9
MW9	10	MN418256	Australia	*Ostreopsis* sp. 9
MW10	10	MN418257	Australia	*Ostreopsis* sp. 9
BH3	10	MN418260	Australia	*Ostreopsis* sp. 9
IFR-OSTS-C9	11	MT952066	Spain/France	*Ostreopsis* sp. 9
IFR-OSTS-C10	11	MT952067	Spain/France	*Ostreopsis* sp. 9
IFR-OSTS-D3	11	MT952020	Spain/France	*Ostreopsis* sp. 9
IFR-OSTS-D8	11	MT952025	Spain/France	*Ostreopsis* sp. 9
IFR-OSTS-D12	11	MT952029	Spain/France	*Ostreopsis* sp. 9
IFR-OSTS-H3	11	MT952039	Spain/France	*Ostreopsis* sp. 9
IFR-OSTS-H4	11	MT952040	Spain/France	*Ostreopsis* sp. 9
IFR-OSTS-H8	11	MT952044	Spain/France	*Ostreopsis* sp. 9
IFR-OSTS-H10	11	MT952046	Spain/France	*Ostreopsis* sp. 9
IFR-OSTS-H13	11	MT952049	Spain/France	*Ostreopsis* sp. 9
IFR-OSTS-SB1	11	MT952006	Spain/France	*Ostreopsis* sp. 9
IFR-OSTS-SB5	11	MT952009	Spain/France	*Ostreopsis* sp. 9
IFR-OSTS-SC5	11	MT952012	Spain/France	*Ostreopsis* sp. 9
IFR-OSTS-SD6	11	MT952018	Spain/France	*Ostreopsis* sp. 9
IFR-OSTS-SD5		MT952017	Spain/France	*Ostreopsis* sp. 9
IFR-OSTS-D4	12	MT952021	Spain/France	*Ostreopsis* sp. 9

IFR-OSTS-D7	12	MT952024	Spain/France	*Ostreopsis* sp. 9
IFR-OSTS-D10	12	MT952027	Spain/France	*Ostreopsis* sp. 9
IFR-OSTS-D11	12	MT952028	Spain/France	*Ostreopsis* sp. 9
IFR-OSTS-H2	12	MT952038	Spain/France	*Ostreopsis* sp. 9
IFR-OSTS-H5	12	MT952041	Spain/France	*Ostreopsis* sp. 9
IFR-OSTS-H6	12	MT952042	Spain/France	*Ostreopsis* sp. 9
IFR-OSTS-H7	12	MT952043	Spain/France	*Ostreopsis* sp. 9
IFR-OSTS-SD4	12	MT952016	Spain/France	*Ostreopsis* sp. 9
IFR-OSTS-C3	13	MT952060	Spain/France	*Ostreopsis* sp. 9
IFR-OSTS-D3	13	MT952015	Spain/France	*Ostreopsis* sp. 9
IFR-OSTS-SB4		MT952008	Spain/France	*Ostreopsis* sp. 9
IFR-OSTS-SC2	14	MT952014	Spain/France	*Ostreopsis* sp. 9
IFR-OSTS-SD2	14	MT952011	Spain/France	*Ostreopsis* sp. 9
IFR-OSTS-D9	15	MT952026	Spain/France	*Ostreopsis* sp. 9
IFR-OSTS-D15	15	MT952032	Spain/France	*Ostreopsis* sp. 9
IFR-OSTS-LA4	15	MT952035	Spain/France	*Ostreopsis* sp. 9
IFR-OSTS-Z1	15	MT952050	Spain/France	*Ostreopsis* sp. 9
IFR-OSTS-Z3	15	MT952052	Spain/France	*Ostreopsis* sp. 9
IFR-OSTS-Z7	15	MT952056	Spain/France	*Ostreopsis* sp. 9
IFR-OSTS-OS3E	16	MT952070	Spain/France	*Ostreopsis* sp. 9
IFR-OSTS-MC3	16	MT952069	Spain/France	*Ostreopsis* sp. 9
IFR-OSTS-C2	16	MT952059	Spain/France	*Ostreopsis* sp. 9
IFR-OSTS-C1	16	MT952058	Spain/France	*Ostreopsis* sp. 9
IFR-OSTS-Z6	16	MT952055	Spain/France	*Ostreopsis* sp. 9
IFR-OSTS-Z4	16	MT952053	Spain/France	*Ostreopsis* sp. 9
IFR-OSTS-Z2	16	MT952051	Spain/France	*Ostreopsis* sp. 9
IFR-OSTS-H12	16	MT952048	Spain/France	*Ostreopsis* sp. 9
IFR-OSTS-H1	16	MT952037	Spain/France	*Ostreopsis* sp. 9
IFR-OSTS-LD3	16	MT952036	Spain/France	*Ostreopsis* sp. 9
IFR-OSTS-D17	16	MT952034	Spain/France	*Ostreopsis* sp. 9
IFR-OSTS-D14	16	MT952031	Spain/France	*Ostreopsis* sp. 9
IFR-OSTS-D13	16	MT952030	Spain/France	*Ostreopsis* sp. 9
IFR-OSTS-D5	16	MT952022	Spain/France	*Ostreopsis* sp. 9
IFR-OSTS-D2	16	MT952019	Spain/France	*Ostreopsis* sp. 9
IFR-OSTS-SC1	16	MT952010	Spain/France	*Ostreopsis* sp. 9
IFR-OSTS-SC6	16	MT952013	Spain/France	*Ostreopsis* sp. 9
IFR-OSTS-SB3	16	MT952007	Spain/France	*Ostreopsis* sp. 9
IFR-OSTS-C6	16	MT952063	Spain/France	*Ostreopsis* sp. 9
IFR-OSTS-Z5	16	MT952054	Spain/France	*Ostreopsis* sp. 9
IFR-OSTS-C11	16	MT952068	Spain/France	*Ostreopsis* sp. 9
IFR-OSTS-C8	16	MT952065	Spain/France	*Ostreopsis* sp. 9
IFR-OSTS-C5	16	MT952062	Spain/France	*Ostreopsis* sp. 9
IFR-OSTS-C4	16	MT952061	Spain/France	*Ostreopsis* sp. 9
IFR-OSTS-D16	16	MT952033	Spain/France	*Ostreopsis* sp. 9
IFR-OSTS-H9	16	MT952045	Spain/France	*Ostreopsis* sp. 9
IFR-OSTS-C7	16	MT952064	Spain/France	*Ostreopsis* sp. 9
IFR-OSTS-D6		MT952023	Spain/France	*Ostreopsis* sp. 9
GB23		MN418303	Australia	*Ostreopsis* sp. 9
Dn86EHU		JX987675	Portugal: Galea	*Ostreopsis* sp. 9
KM2		MN418305	Australia	*Ostreopsis* sp. 9
BH8		MN418265	Australia	*Ostreopsis* sp. 9
KM4	17	MN418307	Australia	*Ostreopsis* sp. 9
KM7	17	MN418310	Australia	*Ostreopsis* sp. 9
KM8	17	MN418311	Australia	*Ostreopsis* sp. 9
KM5	18	MN418308	Australia	*Ostreopsis* sp. 9
KM6	18	MN418309	Australia	*Ostreopsis* sp. 9

MW4	19	MN418251	Australia	*Ostreopsis* sp. 9
MW5	19	MN418252	Australia	*Ostreopsis* sp. 9
MW6	19	MN418253	Australia	*Ostreopsis* sp. 9
PC11		MN418294	Australia	*Ostreopsis* sp. 9
Dn101EHU		JX987679	Portugal: Galea	*Ostreopsis* sp. 9
KM1		MN418304	Australia	*Ostreopsis* sp. 9
143EHU		JX987682	Spain: San Sebastian	*Ostreopsis* sp. 9
PC7	20	MN418290	Australia	*Ostreopsis* sp. 9
PC8	20	MN418291	Australia	*Ostreopsis* sp. 9
PC9	20	MN418292	Australia	*Ostreopsis* sp. 9
PC10	20	MN418293	Australia	*Ostreopsis* sp. 9
PC13	20	MN418295	Australia	*Ostreopsis* sp. 9
LM8	20	MN418285	Australia	*Ostreopsis* sp. 9
LM9	20	MN418286	Australia	*Ostreopsis* sp. 9
LM10	20	MN418287	Australia	*Ostreopsis* sp. 9
MER14	20	MN418312	Australia	*Ostreopsis* sp. 9
MER19	20	MN418313	Australia	*Ostreopsis* sp. 9
CAWD96	20	AB674915	New Zealand: Kerikeri	*Ostreopsis* sp. 9
CAWD203	20	KT868527	Australia: Tasman Sea, Merimbula	*Ostreopsis* sp. 9
MW1		MN418248	Australia	*Ostreopsis* sp. 9
BH4		MN418261	Australia	*Ostreopsis* sp. 9
PC3		MN418288	Australia	*Ostreopsis* sp. 9
GB5	21	MN418298	Australia	*Ostreopsis* sp. 9
GB8	21	MN418299	Australia	*Ostreopsis* sp. 9
**IFR-OST-02E**	**22**	**ON980638**	**France: Saint-Jean-de-Luz**	*Ostreopsis* sp. 9
**IFR-OST-03E**	**22**	**ON980639**	**France: Saint-Jean-de-Luz**	*Ostreopsis* sp. 9
**IFR21-332**	**22**	**ON980621**	**France: Saint-Jean-de-Luz**	*Ostreopsis* sp. 9
**IFR21-333**	**22**	**ON980622**	**France: Saint-Jean-de-Luz**	*Ostreopsis* sp. 9
**IFR21-337**	**22**	**ON980623**	**France: Saint-Jean-de-Luz**	*Ostreopsis* sp. 9
**IFR22-008**	**22**	**ON980624**	**France: Saint-Jean-de-Luz**	*Ostreopsis* sp. 9
**IFR22-014**	**22**	**ON980625**	**France: Saint-Jean-de-Luz**	*Ostreopsis* sp. 9
**IFR22-017**	**22**	**ON980626**	**France: Saint-Jean-de-Luz**	*Ostreopsis* sp. 9
**IFR22-021**	**22**	**ON980627**	**France: Saint-Jean-de-Luz**	*Ostreopsis* sp. 9
**IFR22-024**	**22**	**ON980628**	**France: Saint-Jean-de-Luz**	*Ostreopsis* sp. 9
**IFR22-030**	**22**	**ON980629**	**France: Saint-Jean-de-Luz**	*Ostreopsis* sp. 9
**IFR22-034**	**22**	**ON980630**	**France: Saint-Jean-de-Luz**	*Ostreopsis* sp. 9
**IFR22-037**	**22**	**ON980631**	**France: Saint-Jean-de-Luz**	*Ostreopsis* sp. 9
**IFR22-041**	**22**	**ON980632**	**France: Saint-Jean-de-Luz**	*Ostreopsis* sp. 9
**IFR22-046**	**22**	**ON980633**	**France: Saint-Jean-de-Luz**	*Ostreopsis* sp. 9
**IFR22-056**	**22**	**ON980634**	**France: Saint-Jean-de-Luz**	*Ostreopsis* sp. 9
**IFR22-066**	**22**	**ON980635**	**France: Saint-Jean-de-Luz**	*Ostreopsis* sp. 9
**IFR22-073**	**22**	**ON980636**	**France: Saint-Jean-de-Luz**	*Ostreopsis* sp. 9
PC4	22	MN418289	Australia	*Ostreopsis* sp. 9
GB15	22	MN418301	Australia	*Ostreopsis* sp. 9
GB16	22	MN418302	Australia	*Ostreopsis* sp. 9
MER24	22	MN418315	Australia	*Ostreopsis* sp. 9
GB14	22	MN418300	Australia	*Ostreopsis* sp. 9
Dn144EHU	22	JX987683	Spain: San Sebastian	*Ostreopsis* sp. 9
142EHU	22	JX987681	Spain: San Sebastian	*Ostreopsis* sp. 9
GB3	22	MN418297	Australia	*Ostreopsis* sp. 9
Dn91EHU	22	JX987676	Portugal: Lagos	*Ostreopsis* sp. 9
Dn201EHU	22	JX987699	France: Saint Jean de Luz	*Ostreopsis* sp. 9
Dn188EHU	22	JX987692	Spain: Santona	*Ostreopsis* sp. 9
Dn93EHU	22	JX987678	Portugal: Cascais	*Ostreopsis* sp. 9
Dn160EHU	22	JX987689	Spain: Santona	*Ostreopsis* sp. 9
Dn158EHU	22	JX987688	Spain: Zierbana	*Ostreopsis* sp. 9

Dn192EHU	22	JX987696	Spain: Zierbana	*Ostreopsis* sp. 9
Dn171EHU	22	JX987690	France: Saint Jean de Luz	*Ostreopsis* sp. 9
Dn172EHU	22	JX987691	France: Saint Jean de Luz	*Ostreopsis* sp. 9
Dn189EHU	22	JX987693	Spain: San Sebastian	*Ostreopsis* sp. 9
Dn190EHU	22	JX987694	Spain: San Sebastian	*Ostreopsis* sp. 9
Dn191EHU	22	JX987695	Spain: San Sebastian	*Ostreopsis* sp. 9
Dn92EHU	22	JX987677	Portugal: Galea	*Ostreopsis* sp. 9
Dn196EHU	22	JX987697	Portugal: Galea	*Ostreopsis* sp. 9
MER20	22	MN418314	Australia	*Ostreopsis* sp. 9
CNR-T5	22	FM244729	Italy:Mediterranean, Ionian Sea, Taormina	*Ostreopsis* sp. 9
CNR-B4	22	AJ301643	Italy:Ganzirri, Tyrrhenian Sea	*Ostreopsis* sp. 9
CSIC-D5	22	AJ312944	Spain:Catalon Coast	*Ostreopsis* sp. 9
IEO-1V	22	AJ319871	Spain:Almeria,Andalusia	*Ostreopsis* sp. 9
IEO-2V	22	AJ491313	Spain:Almeria, Andalusia	*Ostreopsis* sp. 9
IEO-3V	22	AJ491332	Spain:Andalusia, Almeria	*Ostreopsis* sp. 9
GB2	22	MN418296	Australia	*Ostreopsis* sp. 9
CNR-B5	22	AJ491335	Italy:Sicily, Tyrrehenian Sea, Ganzirri	*Ostreopsis* sp. 9
CSIC-D1	22	AJ491333	Spain:Catalan coast, La Fosca	*Ostreopsis* sp. 9
CSIC-D7	22	AJ491334	Spain:Catalan Coast, La Fosca	*Ostreopsis* sp. 9
Dn199EHU	22	JX987698	Portugal: Galea	*Ostreopsis* sp. 9
IFR-OSTS-H11	22	MT952047	Spain/France	*Ostreopsis* sp. 9
LM7	23	MN418284	Australia	*Ostreopsis* sp. 9
KM3	23	MN418306	Australia	*Ostreopsis* sp. 9
LM1	24	MN418278	Australia	*Ostreopsis* sp. 9
LM2	24	MN418279	Australia	*Ostreopsis* sp. 9
LM3	24	MN418280	Australia	*Ostreopsis* sp. 9
LM4	24	MN418281	Australia	*Ostreopsis* sp. 9
LM5	24	MN418282	Australia	*Ostreopsis* sp. 9
LM6	24	MN418283	Australia	*Ostreopsis* sp. 9
FR4		MN418271	Australia	*Ostreopsis* sp. 9
BH2	25	MN418259	Australia	*Ostreopsis* sp. 9
BH6	25	MN418263	Australia	*Ostreopsis* sp. 9
BH7	25	MN418264	Australia	*Ostreopsis* sp. 9
BH10	25	MN418267	Australia	*Ostreopsis* sp. 9
FR1	25	MN418268	Australia	*Ostreopsis* sp. 9
FR2	25	MN418269	Australia	*Ostreopsis* sp. 9
FR3	25	MN418270	Australia	*Ostreopsis* sp. 9
FR5	25	MN418272	Australia	*Ostreopsis* sp. 9
FR6	25	MN418273	Australia	*Ostreopsis* sp. 9
FR9	25	MN418276	Australia	*Ostreopsis* sp. 9
FR10	25	MN418277	Australia	*Ostreopsis* sp. 9
FR7	26	MN418274	Australia	*Ostreopsis* sp. 9
FR8	26	MN418275	Australia	*Ostreopsis* sp. 9
BH5		MN418262	Australia	*Ostreopsis* sp. 9
HER21		KX055875	Australia : Heron Reef (Queensland)	*O. rhodesiae*
HER20		KX055876	Australia : Heron Reef (Queensland)	*O. rhodesiae*
HER25		KX055874	Australia : Heron Reef (Queensland)	*O. rhodesiae*
P-0135		KM032215	France: Reunion Island, W Indian Ocean	*Ostreopsis* sp. 8
P-0121		KM032219	France: Reunion Island, W Indian Ocean	*Ostreopsis* sp. 8
P-0127		KM032214	France: Reunion Island, W Indian Ocean	*Ostreopsis* sp. 8
CAWD184		AB674914	Cook islands	*Ostreopsis* sp. 3
TB38OS		AB841216	Thailand:Phang-Nga, Khao Lak	*Ostreopsis* sp. 7
TB34OS		AB841214	Thailand:Phang-Nga, Khao Lak	*Ostreopsis* sp. 7
CBA C1019		LN875552	Cyprus, Aegean Sea, Mediterranean	*O. fattorussoi*
VGO881		FM244637	Spain:East Atlantic, Canary Island, Lanzarote	*O. fattorussoi*

CBA C1005	LT220222	Cyprus, Aegean Sea, Mediterranean	*O. fattorussoi*
CBA C1012	LN875554	Cyprus, Aegean Sea, Mediterranean	*O. fattorussoi*
CBA L1020	LT220224	Lebanon, Batroun	*O. fattorussoi*
CAWD179	AB674916	Australia	*O. fattorussoi*
VNPQ218	MT968510	Viet Nam	*O. siamensis*
PNA19-6	MT183168	Tahiti, French Polynesia	*O. siamensis*
TF29OS	AB841255	Thailand:Trat, Koh Wai	*O. siamensis*
