# Supplementary material for: First Characterization of Ostreopsis cf. ovata (Dinophyceae) and Detection of Ovatoxins during a Multispecific and Toxic Ostreopsis Bloom on French Atlantic Coast
Source: Mar Drugs. 2022 Jul 18;20(7):461. doi: 10.3390/md20070461 (PMC9315632; doi:10.3390/md20070461)
Supplement: Supplementary file 1 [file marinedrugs-20-00461-s001.zip › Table S2.pdf]

Table S2. Molecular ion transitions (Q1> Q3) and wavelengths used for the detection of palytoxin and its analogues (ovatoxins, ostreocins, mascarenotoxins)

Analogs	Q1 (*m/z*)	Q3 (*m/z*, fragment A)	UV detection λ (nm)	References
PlTX	1340.2 $[M+2H]^{2+}$<br>1331.2 $[M+2H-H_2O]^{2+}$<br>887.5 $[M+3H-H_2O]^{3+}$	327.2	233, 263	[31, 93]
42-OH-PlTX	1348.2 $[M+2H]^{2+}$<br>1339.2 $[M+2H-H_2O]^{2+}$<br>899.7 $[M+3H]^{3+}$	327.2	233, 263	[94, 95]
Ovatoxin-a	1324.3 $[M+2H]^{2+}$<br>1315.2 $[M+2H-H_2O]^{2+}$<br>876.8 $[M+3H-H_2O]^{3+}$	327.2	233, 263	[31, 40]
Ovatoxin-b	1346.3 $[M+2H]^{2+}$<br>1337.3 $[M+2H-H_2O]^{2+}$<br>891.5 $[M+3H-H_2O]^{3+}$	371.2	233, 263	[33, 40]
Ovatoxin-c	1354.3 $[M+2H]^{2+}$<br>1345.3 $[M+2H-H_2O]^{2+}$<br>896.8 $[M+3H-H_2O]^{3+}$	371.2	233, 263	[33, 40]
Ovatoxin-d	1332.3 $[M+2H]^{2+}$<br>1323.2 $[M+2H-H_2O]^{2+}$<br>882.2 $[M+3H-H_2O]^{3+}$	327.2	233, 263	[33, 40]
Ovatoxin-e	1332.3 $[M+2H]^{2+}$<br>1323.2 $[M+2H-H_2O]^{2+}$<br>882.2 $[M+3H-H_2O]^{3+}$	343.2	233, 263	[33, 40]
Ovatoxin-f	1338.3 $[M+2H]^{2+}$<br>1329.3 $[M+2H-H_2O]^{2+}$<br>886.2 $[M+3H-H_2O]^{3+}$	327.2	233, 263	[34, 40]
Ovatoxin-g	1316.3 $[M+2H]^{2+}$<br>1307.2 $[M+2H-H_2O]^{2+}$<br>871.5 $[M+3H-H_2O]^{3+}$	327.2	233, 263	[35]
Ovatoxin-h	1317.3 $[M+2H]^{2+}$<br>1308.3 $[M+2H-H_2O]^{2+}$<br>872.2 $[M+3H-H_2O]^{3+}$	327.2	233, 263	[36]
Ovatoxin-i	1345.3 $[M+2H]^{2+}$<br>1336.3 $[M+2H-H_2O]^{2+}$<br>890.8 $[M+3H-H_2O]^{3+}$	327.2	233, 263	[40]
Ovatoxin-j1/j2	1353.3 $[M+2H]^{2+}$<br>1344.3 $[M+2H-H_2O]^{2+}$<br>896.2 $[M+3H-H_2O]^{3+}$	327.2	233, 263	[40]
Ovatoxin-k	1361.3 $[M+2H]^{2+}$<br>1352.2 $[M+2H-H_2O]^{2+}$<br>901.5 $[M+3H-H_2O]^{3+}$	327.2	233, 263	[40]
Ostreocin-D	1329.2 $[M+H+Na]^{2+}$<br>1318.2 $[M+2H]^{2+}$<br>893.3 $[M+H+2Na]^{3+}$	313.2	233, 263	[44]
Ostreocin-B	1337.2 $[M+H+Na]^{2+}$<br>1326.2 $[M+2H]^{2+}$<br>898.7 $[M+H+2Na]^{3+}$	313.2	233, 263	[47, 80]
Ostreocin-A	1337.2 $[M+H+Na]^{2+}$<br>1326.2 $[M+2H]^{2+}$<br>898.7 $[M+H+2Na]^{3+}$	313.2	233, 263	[46]
Ostreocin-E1	1320.2 $[M+H+Na]^{2+}$<br>1309.2 $[M+2H]^{2+}$<br>887.3 $[M+H+2Na]^{3+}$	313.2	233, 263	[46]
Mascarenotoxin-A	1295.5<br>836.9<br>606.3	327.2	233, 263	[96, 97]
Mascarenotoxin-B	1304.3<br>864.9<br>836.2	327.2	233, 263	[96]
Mascarenotoxin-C	1326.3 $[M+H+Na]^{2+}$<br>1315.3 $[M+2H]^{2+}$<br>877 $[M+3H]^{3+}$	327.2	233, 263	[97]
