# Supplementary material for: First Characterization of Ostreopsis cf. ovata (Dinophyceae) and Detection of Ovatoxins during a Multispecific and Toxic Ostreopsis Bloom on French Atlantic Coast
Source: Mar Drugs. 2022 Jul 18;20(7):461. doi: 10.3390/md20070461 (PMC9315632; doi:10.3390/md20070461)
Supplement: Supplementary file 1 [file marinedrugs-20-00461-s001.zip › Table S3.pdf]

Table S3. Summary of samples collected for the different analyses

Samples collected				Type of analysis			
**Site**	**Date**	**water**	**macroalga**	**Cell counts**	**Molecular id.**	**Toxins**	**Strain isolation**
Hendaye	18-Sep-20	-	*Cystoseira tamariscifolia* .	+	-	-	-
Erromardie	03-Aug-21	+	*Gelidium corneum*	+	-	-	-
Erromardie	09-Aug-21	+	*Gelidium corneum, Halopithys incurva*	+	-	-	-
Erromardie	16-Aug-21	+	-	+	+	+	-
Erromardie	24-Aug-21	+	*Gelidium corneum*	+	-	-	-
Erromardie	01-Sep-21	+	-	+	-	-	-
Erromardie	07-Sep-21	+	*Gelidium corneum*	+	-	-	+
Erromardie	20-Sep-21	+	*Gelidium corneum*	+	-	-	-
Viviers Basques	09-Aug-21	+	*Gelidium corneum*	+	-	-	-
Viviers Basques	24-Aug-21	+	*Gelidium corneum*	+	-	-	-
Viviers Basques	07-Sep-21	+	*Gelidium corneum*	+	-	-	-
Viviers Basques	20-Sep-21	+	*Pterocladiella capillacea*	+	-	-	-
Parlementia	09-Aug-21	+	*Gelidium corneum*	+	-	-	-
Parlementia	24-Aug-21	+	*Gelidium corneum*	+	-	-	-
Parlementia	07-Aug-21	+	*Gelidium corneum*	+	-	-	-
Parlementia	20-Aug-21	+	*Gelidium corneum*	+	-	-	-
